# Single mutations toggle the substrate selectivity of multifunctional *Camptotheca* secologanic acid synthases

**DOI:** 10.1016/j.jbc.2022.102237

**Published:** 2022-07-06

**Authors:** Justin C. Miller, Mary A. Schuler

**Affiliations:** 1 Department of Chemistry, University of Illinois Urbana-Champaign, Urbana, Illinois, USA; 2 Department of Cell and Developmental Biology, University of Illinois Urbana-Champaign, Urbana, Illinois, USA; 3 Department of Biochemistry, University of Illinois Urbana-Champaign, Urbana, Illinois, USA; 4 Department of Plant Biology, University of Illinois Urbana-Champaign, Urbana, Illinois, USA

**Keywords:** cytochrome P450, enzyme catalysis, enzyme mutation, homology modeling, natural product biosynthesis, plant biochemistry, secondary metabolism, ancestral sequence reconstruction, *Camptotheca acuminata*, 7DLH, 7-deoxyloganic acid hydroxylase, ACN, acetonitrile, ASR, ancestral sequence reconstruction, CYP P450, cytochrome P450, CPR, cytochrome P450 reductase, DLPC, 1,2-dilauroyl-sn-glycero-3-phosphocholine, LAMT, loganic acid *O*-methyltransferase, SLAS, secologanic acid synthase, SLS, secologanin synthase, SRS, substrate recognition sequence, TIA, terpene indole alkaloid

## Abstract

Terpene indole alkaloids (TIAs) are plant-derived specialized metabolites with widespread use in medicine. Species-specific pathways derive various TIAs from common intermediates, strictosidine or strictosidinic acid, produced by coupling tryptamine with secologanin or secologanic acid. The penultimate reaction in this pathway is catalyzed by either secologanin synthase (SLS) or secologanic acid synthase (SLAS) according to whether plants produce secologanin from loganin or secologanic acid from loganic acid. Previous work has identified SLSs and SLASs from different species, but the determinants of selectivity remain unclear. Here, combining molecular modeling, ancestral sequence reconstruction, and biochemical methodologies, we identified key residues that toggle SLS and SLAS selectivity in two CYP72A (cytochrome P450) subfamily enzymes from *Camptotheca acuminata*. We found that the positions of foremost importance are in substrate recognition sequence 1 (SRS1), where mutations to either of two adjacent histidine residues switched selectivity; His131Phe selects for and increases secologanin production whereas His132Asp selects for secologanic acid production. Furthermore, a change in SRS3 in the predicted substrate entry channel (Arg/Lys270Thr) and another in SRS4 at the start of the I-helix (Ser324Glu) decreased enzyme activity toward either substrate. We propose that the *Camptotheca* SLASs have maintained the broadened activities found in a common asterid ancestor, even as the *Camptotheca* lineage lost its ability to produce loganin while the campanulid and lamiid lineages specialized to produce secologanin by acquiring mutations in SRS1. The identification here of the residues essential for the broad substrate scope of SLASs presents opportunities for more tailored heterologous production of TIAs.

Terpene indole alkaloids (TIAs) encompass thousands of specialized metabolites produced by a wide array of Asterids. These compounds, acting as defensive agents against various biotic stresses (herbivory, fungal or bacterial infection, etc.), enable these species to adapt to changing environments. The diversity of TIAs produced, the many species that produce them, the geographic distribution of these plants, and the biological activity of the compounds underlie their use as medicines across cultures and throughout the world.

Often complex in their fused ring systems, position of oxidations, and stereochemistry (Fig. 1), TIAs are not readily synthesized at scales conducive to industrial production (1, 2). The molecules are instead isolated from producing species with variable success owing to their low concentrations in plant materials, environmentally influenced production, and difficulties in purifying the desired compound from the complex matrices in which they accumulate (bark, leaves, fruits, etc.).

**Figure 1:**
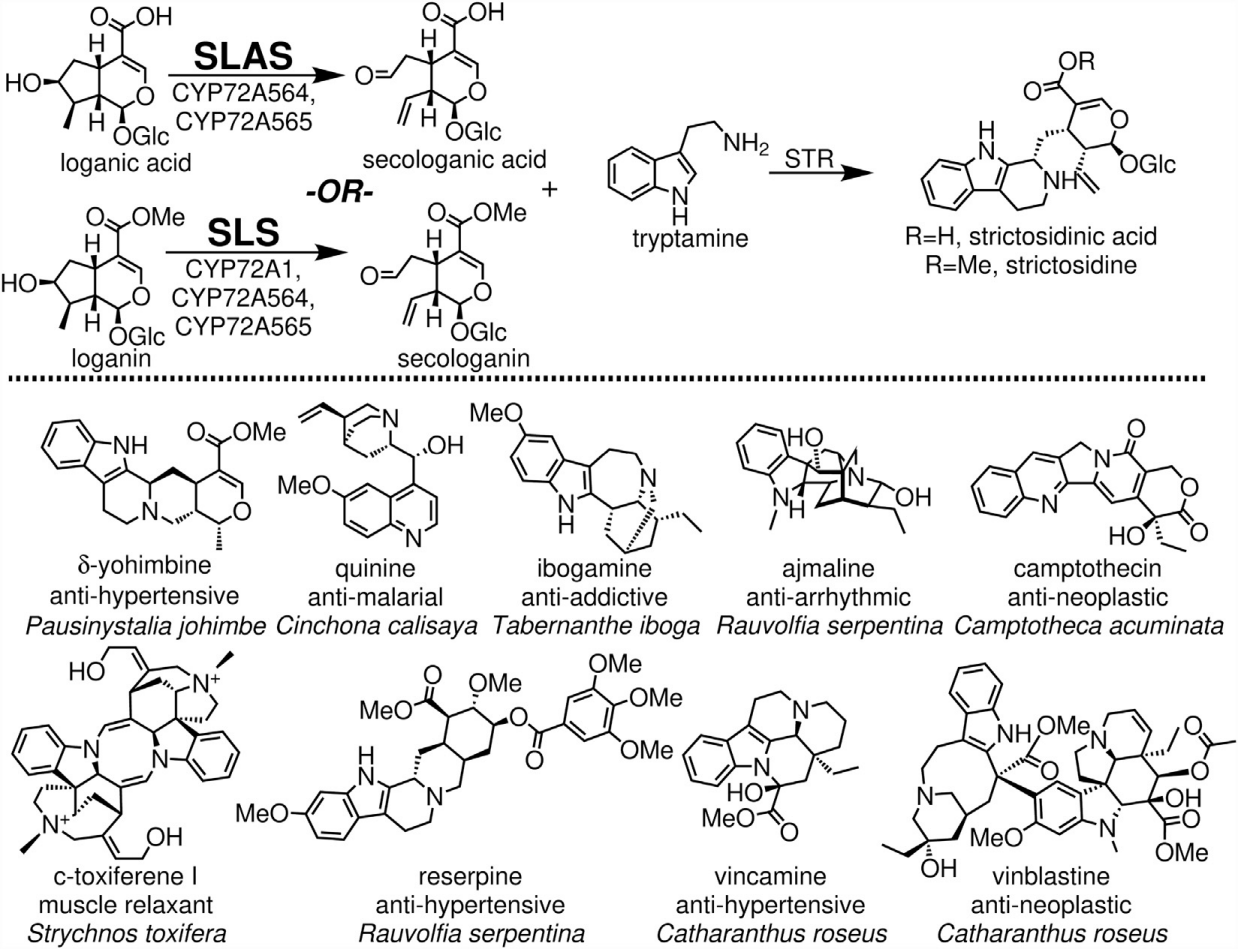
**Production of strictosidinic acid derivatives and the terpene indole alkaloids derived from them.** CYP72As–denoted secologanin synthases (SLSs) and secologanic acid synthases (SLASs) produce secoiridoids from loganin and loganic acid, respectively, that react with tryptamine to form strictosidinic acid derivatives. Species-specific pathways then modify these core structures into a variety of biologically active, medicinally useful TIAs. TIA, terpene indole alkaloid.

In spite of the immense structural diversity of TIAs among medicinal plants, a relatively common biosynthetic pathway exists wherein a secoiridoid is coupled to tryptamine to produce a common strictosidinic acid core (Fig. 1). This shared biosynthetic system provides an avenue for heterologous production of TIAs to alleviate the limited productivity of harvesting from native producers. The elucidation of the biosynthetic genes from *Catharanthus roseus* (3, 4, 5, 6, 7, 8, 9, 10, 11, 12, 13, 14), for example, inspired the production of strictosidine in *Saccharomyces cerevisiae* (15). Though able to isolate strictosidine, analyses of these cultures found large amounts of loganin—an indication that the cytochrome P450 (CYP) secologanin synthase (SLS) was limiting TIA production.

Though SLSs have been characterized from multiple species (*Lonicera japonica* (16), *C. roseus* (4, 5), *Nothapodytes nimmoniana* (17), and *Ophiorrhiza pumila* (18)) with multiple isoforms (5, 15, 18), the difficulties of working with membrane-bound CYPs have complicated biochemical characterizations aimed at optimizing the enzymatic activities of any SLS. Moreover, the lack of studies reporting SLS activity outside of microbial cultures (4, 15), lysates (4, 5), or microsomal fractions (4, 16, 17) suggests that the best-studied *Catharanthus* SLS isoforms suffer from poor expression and/or protein instability in heterologous hosts. The recent identification (19, 20) of two secologanic acid synthases (SLASs) from *Camptotheca acuminata* that are also active as SLSs provides a set of secologanin-producing enzymes that may serve as alternatives to the problematic *Catharanthus* SLSs. That these *Camptotheca* CYPs are amenable to heterologous expression, purification, and biochemical assessment (20) suggests that they are more stable in heterologous systems than any of the *Catharanthus* SLS variants.

The ability of these versatile *Camptotheca* CYPs to produce secologanic acid from loganic acid as well as secologanin from loganin, however, presents a key hurdle for the efficient production of strictosidine in heterologous species. We therefore undertook the identification of residues enabling the differentiation of loganic acid and loganin and, thereby, SLAS versus SLS activity. For this venture, homology modeling and ancestral sequence reconstruction (ASR) were combined to identify sites likely involved in substrate recognition by the various SLSs/SLASs. Site-directed mutagenesis of these sites in the *Camptotheca* SLASs and subsequent *in vitro* assays confirmed that mutation at any of the four sites perturbed substrate binding and/or turnover for loganic acid and loganin. We also assessed the concerted effect of these mutations by expressing the predicted SLS, SLAS common ancestor. Importantly, for future attempts aimed at improving secologanin production in heterologous systems, one key mutation in these *Camptotheca* SLASs improved loganin binding and turnover while abolishing loganic acid binding and turnover.

## Results

### ASR identifies four loci in substrate recognition sequences

Our previous work with homology models (20) identified the conserved His132 of *Camptotheca* CYP72A564 and CYP72A565 as likely important for these enzymes’ ability to turnover loganic acid. Cognizant that these models are but snapshots of dynamic proteins, we determined to apply another methodology to identify residues effecting substrate differentiation between loganic acid and loganin.

The publication of multiple medicinal plant genomes over the past decade presented sufficient numbers of sequences for ASR—a method with several advantages for our endeavors: first, by including putative SLS proteins from species besides *Catharanthus*, ASR had potential to remove the focus from variations particular to just SLS sequences from *Catharanthus*; second, the relative speed and computational expenses of ASR allowed the evaluation of more sequences than possible with molecular dynamics (MD) simulations; third, unlike our previously developed static models (20), ASR had potential to identify residues important for substrate differentiation not immediately adjacent to the heme cofactor.

**Figure 2:**
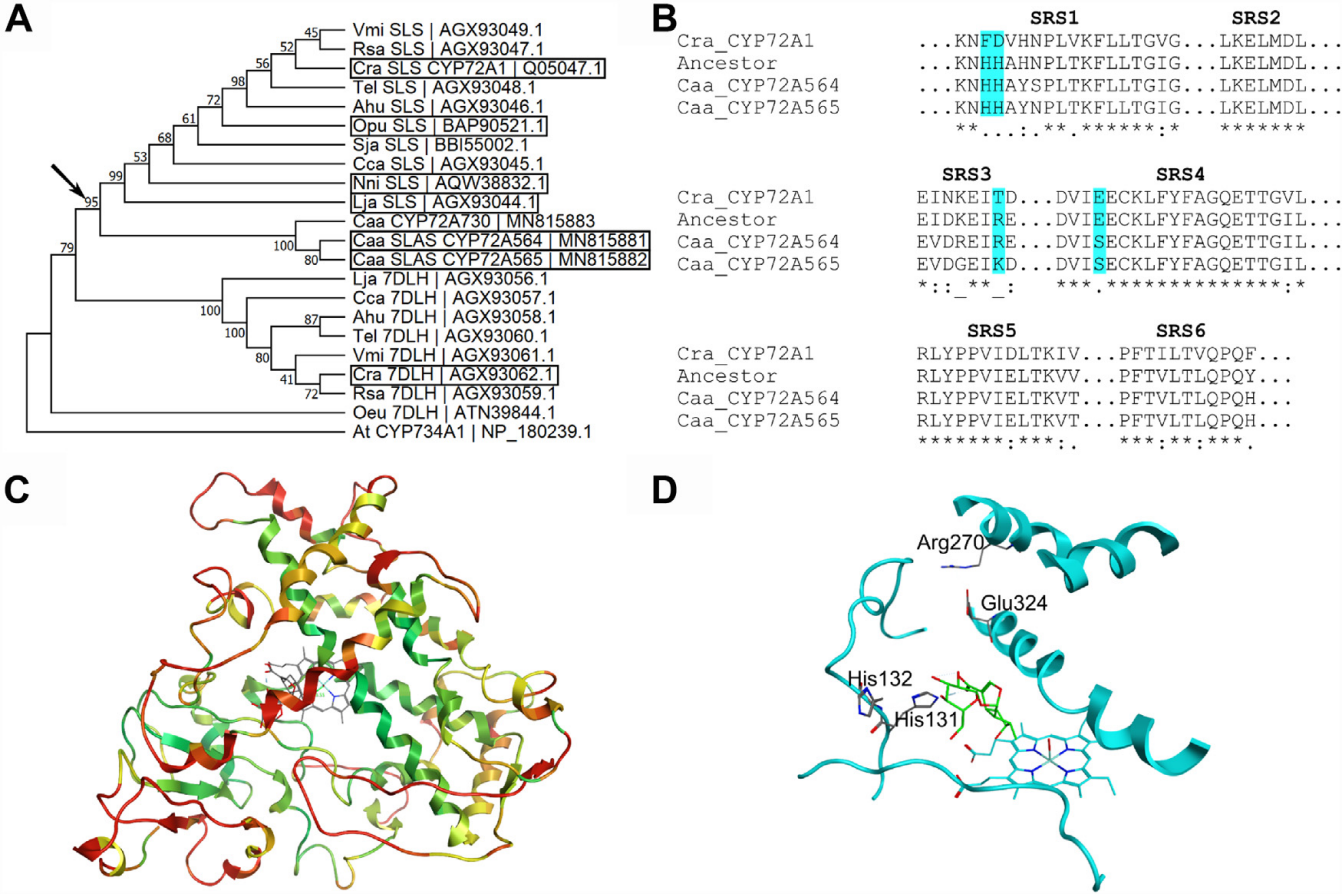
**Ancestral sequence reconstruction of the secologanin, secologanic acid synthase common ancestor.***A*, maximum likelihood, bootstrap consensus tree of CYP72As annotated as SLS, 7-deoxyloganic acid hydroxylase (7DLH), SLS-like, or 7DLH-like from strictosidine-producing species used for the ASR. The evolutionary history was inferred using the model of Le and Gascuel (46) with a discrete Gamma distribution using MEGA X (44). Genes surrounded by a box denote those for which the given activity has been reported in the literature (4, 5, 7, 16, 17, 18, 19, 20). Abbreviations and descriptions for the genes are located in Table S1. *B*, multiple sequence alignment of the predicted substrate recognition sequences (SRSs) comparing the *Catharanthus* CYP72A1, *Camptotheca* CYP72A564 and CYP72A565, and the predicted SLS, SLAS common ancestor. *Cyan* boxes indicate residues mutated in the *Camptotheca* enzymes for analysis. *C*, ribbon cartoon of the SLS, SLAS common ancestor docked with loganic acid. Coloration corresponds to the average backbone RMSD of the ancestor compared to models for *Camptotheca* CYP72A564 and CYP72A565: *green*, 0.0 Å; *yellow*, 2.0 Å; *red*, 4.0 Å. *D*, SLS, SLAS common ancestor homology model docked with loganic acid (*green*) with the predicted SRSs (*cyan* ribbon cartoon) and the four amino acids selected for mutation in the *Camptotheca* enzymes shown. 7DLH, 7-deoxyloganic acid hydroxylase; ASR, ancestral sequence reconstruction; SLS, secologanin synthase.

Sequences annotated as SLS, 7-deoxyloganic acid hydroxylase (7DLH), SLS-like, or 7DLH-like within GenBank from species known to produce secoiridoids (Table S1) were used to construct the phylogenetic tree (Fig. 2*A*). We used only sequences from species known to produce secoiridoids recognizing that SLSs and 7DLHs are only a small subset of the enzymes within the very large CYP72A subfamily (21). For several reasons, the inclusion of 7DLHs was important to determine a common SLS, SLAS ancestor. First, these SLSs, SLASs, and 7DLHs are all members of the CYP72A subfamily, have upward of 80% homology, and utilize highly similar substrates. Second, the *Camptotheca* SLASs have demonstrated all three activities—7DLH, SLS, and SLAS (19)—presenting the possibility that they are more similar to extant 7DLHs than to SLSs. Third, only six of the sequences annotated as SLS (4, 5, 16, 17, 18) or 7DLH (7) in GenBank have been characterized biochemically.

After a multiple-sequence alignment that also included *Arabidopsis* CYP734A1 (a member of the closest subfamily to CYP72A (21)) to root the tree, we constructed a maximum likelihood bootstrap consensus tree (Fig. 2*A*) that was predominantly in agreement with the established phylogenetic relationships among the included species (22). The SLS, SLAS lineage separates Cornales (*Camptotheca*, Caa) from the campanulids (Dipsacales: *Lonicera*, Lja; Aquifoliales II: *Nothapodytes*, Nni) and the lamiids (Gentianales: *Cinchona*, Cca; *Swertia*, Sja; *Ophiorrhiza*, Opu; *Amsonia*, Ahu; *Tabernaemontana*, Tel; *Catharanthus*, Cra; *Rauvolfia*, Rsa; *Vinca*, Vmi). *Swertia* (Gentianaceae), however, does intercalate between two constituents of Rubiaceae (*Cinchona* and *Ophiorrhiza*)—albeit without a strong bootstrap value (61, Fig. 2*A*). The putative 7DLH from *Olea* (Oeu 7DLH) did not group with either the SLS or 7DLH lineage in spite the large number of lamiids represented. Several other CYP72As from *Olea* with considerable homology to *Catharanthus* SLS are known to metabolize secoiridoids (23), supporting the possibility that this 7DLH-like gene may be among the many CYP72As that mediate other reactions. Notwithstanding these caveats, we retained this phylogenetic tree for the prediction of a common SLS, SLAS ancestor because they did not affect the common ancestor of *Camptotheca* and the core asterids.

Focusing on the SLS, SLAS ancestor (node 34, Figs. S1 and S2), we began by comparing the predicted substrate recognition sequences (SRSs) of this ancestral protein to those of the *Camptotheca* SLASs and *Catharanthus* SLS (Figs. 2*B*, S3, and File S1). In addition to the previously implicated His132 (20) (numbered according to the *Camptotheca* SLASs), a neighboring His131 in SRS1, Arg/Lys270 in SRS3, and Ser324 in SRS4 appeared as sites of change between the SLS, SLAS ancestor and extant sequences. We then constructed a homology model (Figs. 2, *C* and *D* and S4) of the putative SLS, SLAS ancestor with loganic acid docked into the active site in order to visualize these sites and infer their importance.

With our previous predictions (20) suggesting that His132 in the *Camptotheca* SLASs interacts with the carboxylic acid moiety of loganic acid and that Asp132 in nearly all SLSs (File S1) prevents favorable interactions, we predicted that a His132Asp mutation would logically prevent loganic acid binding and turnover completely. Facing outward from the predicted active site in the extant *Camptotheca* SLASs’ and *Catharanthus* SLS’s homology models but a contact in the SLS, SLAS ancestor’s homology model, His131 (Phe in all the SLSs) arose as another residue of probable importance. We predicted that disrupting a possible hydrogen bond donor to and electrostatic contact with loganic acid would negatively affect binding and turnover of loganic acid without affecting these enzymes’ use of loganin.

The reasons why the models for the extant SLASs and the predicted ancestral sequence differ so drastically in their placement of these adjacent His residues remains unclear. The SLS, SLAS ancestor has two fewer amino acids, but a multiple sequence alignment (Fig. S3 and File S1) places the subsequent gap in the signal anchor domain—a portion of the protein not included in the homology model. The template crystal structure, sequence alignment parameters, force fields, and docking methodologies used to generate the three homology models were all identical. With the exception of SRS3 along the G-helix (Fig. 2*C*), the extant *Camptotheca* SLASs’ and the SLS, SLAS ancestor’s homology models have little backbone variation as measured by RMSD. Lacking a definitive model for differentiating whether His131 or His132 is of greater importance, we constructed single and double mutants at both of these sites. We anticipated that mutations at one of the sites would produce a strong effect, mutations at the other would have a weak effect (if any), and mutation at both sites would produce the same effect as that of the stronger of the two single mutants.

ASR identified two other sites not highlighted in our previous homology models. The first of these (Arg/Lys270) in the G-helix, though distant from the site of oxidation within the enzyme, points into the substrate entry channel where a basic, cationic residue might facilitate the entry of loganic acid into the active site. We predicted that mutations here might produce only modest effects on loganic acid binding and turnover as favorable interactions lying in the active site would still permit binding.

The second of these (Ser324) is the lone residue within the SRSs at which the SLS, SLAS ancestor differed from both extant *Camptotheca* SLASs; the ancestral sequence has Glu at this position like *Catharanthus* and all other included SLSs (File S1). Relevant to this position, solution NMR experiments on two bacterial CYPs (CYP101A1 (24) and MycG (25)) have demonstrated that the end of the I-helix at which Ser324 sits in the *Camptotheca* SLASs serves as a hinge allowing the protein to open and close for substrate binding. The homology model of the SLS, SLAS ancestor includes more H-bonding interactions around its Glu than the Ser of CYP72A564 or CYP72565 in their models (Fig. S5), perhaps hindering the hinging required to open and close the active site. Though we did not rule out effects on binding, we anticipated that mutating Ser324 to the much larger Glu would slow substrate entry and product exit thereby slowing the turnover rate.

### Mutation at these loci perturbs type I binding, steady-state kinetics

**Figure 3:**
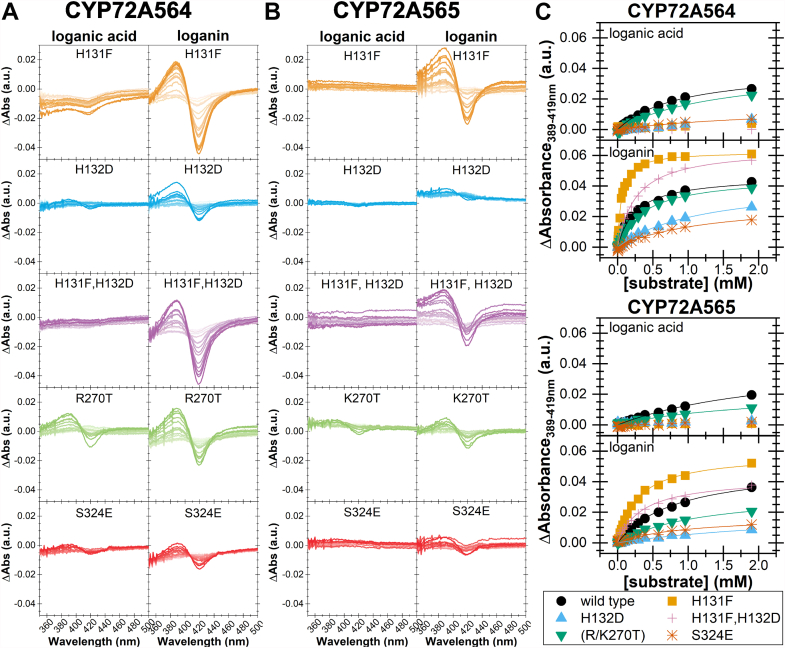
**Type I–binding spectra of *Camptotheca*CYP72A564andCYP72A565mutants.** Difference spectra from 998 nM (*lighter color*) to 1.90 mM (*darker color*) loganic acid or loganin for CYP72A564 (*A*) and CYP72A565 (*B*) mutants. *C*, binding isotherms derived by plotting the difference between the peak (389 nm) and trough (419 nm) of the difference spectra against the substrate concentration using OriginPro 2019. Tables 1, S3, and S4 record the fit parameters.

As indicated by Type I binding spectra (Fig. 3 and Table 1), each of these five mutations affected the binding of loganic acid and loganin to CYP72A564 and CYP72A565. Consistent with our predictions from the ASR and homology modeling, the His132Asp mutant eliminated loganic acid–induced spectral shifts suggesting that the substrate does not bind near the heme iron as required for catalysis. This same mutation also reduced the affinity of these SLASs for loganin as witnessed by the 3.8- to 4.7-fold increase in *K*_*s*_ for CYP72A565 and CYP72A564, respectively (Table 1). These perturbations in substrate binding were reflected in a lack of loganic acid turnover and increased *K*_*M*_ for loganin for both enzymes (Tables 2 and 3). The His132Asp mutant did not greatly affect the *k*_*cat*_ of either CYP resulting in a greater than 5-fold reduction in *k*_*cat*_/*K*_*M*_ for both proteins.

**Table 1:** Type I–binding isotherm parameters of CYP72A564 and CYP72A565 mutants

CYP	Mutation	*K*_s_/*mM*		ΔA_max_/*mA.U.*	
		loganic acid	loganin	loganic acid	loganin
72A564	WT^a^	0.874 ± 0.057^b^	0.256 ± 0.019^b^	39.5 ± 1.3^b^	46.6 ± 1.2^b^
	H131F	n.d.	0.0547 ± 0.0030^c^	n.d.	62.46 ± 0.79^c^
	H132D	n.d.	1.194 ± 0.048^d^	n.d.	42.68 ± 0.97^b^
	H131F,H132D	n.d.	0.258 ± 0.012^b^	n.d.	64.8 ± 1.1^c^
	R270T	1.47 ± 0.36^b,c^	0.3708 ± 0.0081^e^	40.6 ± 6.0^b^	46.62 ± 0.40^b^
	S324E	2.5 ± 1.1^c^	1.30 ± 0.35^d^	15.9 ± 4.7^b^	30.4 ± 4.8^b^
72A565	WT^a^	2.41 ± 0.37^b^	0.841 ± 0.070^b^	43.9 ± 4.6^b^	51.0 ± 2.2^b,c^
	H131F	n.d.	0.283 ± 0.013^c^	n.d.	58.2 ± 1.0^b^
	H132D	n.d.	3.2 ± 1.3^d^	n.d.	22.9 ± 6.5^b,c,d,e^
	H131F,H132D	n.d.	0.345 ± 0.041^c^	n.d.	42.5 ± 2.0^c^
	K270T	2.00 ± 0.61^b^	1.201 ± 0.084^e^	22.4 ± 4.5^b^	33.7 ± 1.3^d^
	S324E	n.d.	0.97 ± 0.25^b,d,e^	n.d.	17.5 ± 2.4^e^

n.d., no data because one or more fit parameters were not significantly different than zero.

Different superscript letters in a column for either CYP denote significantly different fit parameters by comparing 95% confidence intervals calculated *via* an *F*-test (see Tables S3 and S4).

a Data from Miller *et al*. (20).

**Table 2:** Steady state kinetic parameters of CYP72A564 mutants

Substrate	Mutation	*K*_M_	*k*_cat_	*k*_cat_/*K*_M_
		*mM*	*min*^*−1*^	*min*^*−1*^ *mM*^*−1*^
loganic acid	WT^a^	2.67 ± 0.80^b^	13.9 ± 2.1	5.2 ± 1.7
	H131F	n.d.	n.d.	n.d.
	H132D	n.d.	n.d.	n.d.
	H131F,H132D	n.d.	n.d.	n.d.
	R270T	3.54 ± 0.51^b^	20.0 ± 1.6	5.7 ± 0.9
	S324E	n.d.	n.d.	n.d.
loganin	WT^a^	0.24 ± 0.02^b^	12.0 ± 0.3	49.5 ± 4.8
	H131F	0.05 ± 0.01^c^	7.9 ± 0.2	149 ± 18
	H132D	1.44 ± 0.06^d^	13.7 ± 0.3	9.5 ± 0.5
	H131F,H132D	0.38 ± 0.16^b,e^	6.4 ± 0.8	17.1 ± 7.4
	R270T	1.03 ± 0.32^d,e^	19.8 ± 2.5	19.2 ± 6.5
	S324E	1.26 ± 0.10^d^	10.0 ± 0.3	5.2 ± 1.7

n.d., no data because one or more fit parameters were not significantly different than zero.

Different superscript letters in a column for either CYP denote significantly different fit parameters by comparing 95% confidence intervals calculated *via* an *F*-test (see Table S5).

a Data from Miller *et al*. (20).

**Table 3:** Steady state kinetic parameters of CYP72A565 mutants

Substrate	Mutation	*K*_M_	*k*_cat_	*k*_cat_/*K*_M_
		*mM*	*min*^*−1*^	*min*^*−1*^ *mM*^*−1*^
loganic acid	WT^a^	3.57 ± 0.42^b^	19.4 ± 1.3	5.5 ± 0.7
	H131F	n.d.	n.d.	n.d.
	H132D	n.d.	n.d.	n.d.
	H131F,H132D	n.d.	n.d.	n.d.
	K270T	n.d.	n.d.	n.d.
	S324E	n.d.	n.d.	n.d.
loganin	WT^a^	0.72 ± 0.07^b^	15.3 ± 0.5	21.2 ± 2.1
	H131F	0.29 ± 0.08^b^	7.7 ± 0.6	26.7 ± 7.8
	H132D	5.3 ± 2.1^c,d^	20.6 ± 4.9	3.9 ± 1.8
	H131F,H132D	1.71 ± 0.10^c^	31.8 ± 1.4	16.8 ± 1.1
	K270T	1.98 ± 0.74^b,c,d^	10.3 ± 1.8	5.2 ± 2.2
	S324E	3.58 ± 0.68^d^	19.4 ± 2.0	5.4 ± 1.2

n.d., no data because one or more fit parameters were not significantly different than zero.

Different superscript letters in a column for either CYP denote significantly different fit parameters by comparing 95% confidence intervals calculated *via* an *F*-test (see Table S6).

a Data from Miller *et al*. (20).

Mutating the nearby His131 residue to Phe also eliminated loganic acid–induced spectral shifts (Fig. 3). Unlike the His132 mutants, the His131Phe mutation increased the enzymes’ affinity for loganin: a 4.7-fold smaller *K*_*s*_ for CYP72A564 and 3.0-fold smaller *K*_*s*_ for CYP72A565 (Table 1). Once more, the perturbations in Type I binding corresponded with changes in steady state kinetic parameters. Although CYP72A565 His131Phe demonstrated a slightly lower *K*_*M*_ for loganin, the *K*_*M*_ of the same mutant in CYP72A564 was 4.6-fold lower (Table 2). Small but appreciably slower rate constants yielded mutant enzymes with larger specificity constants than the WT with a 3-fold increase for CYP72A564 and a 25% increase for CYP72A565. Neither of the mutants appeared to metabolize loganic acid.

Neither the CYP72A564 nor the CYP72A565 double mutant (His131Phe, His132Asp) demonstrated loganic acid–induced Type I spectra (Fig. 3 and Table 1) nor was there evidence of loganic acid turnover (Tables 2 and 3). When combining the increased binding and turnover of loganin in the His131Phe mutants with the immense loss of these for the His132Asp mutants, the loganin-induced Type I spectra and kinetic parameters of the double mutants with this substrate were roughly the average of those observed with the individual mutations and comparable to those of the WT enzymes. In CYP72A564 for which the effect of His131Phe was larger, neither the *K*_*s*_ nor the *K*_*M*_ in the double mutant differed greatly from the WT (Tables 1 and 2). In CYP72A565 for which the effect of His132Asp was larger, the double mutant’s *K*_*M*_ was more than twice as large as that of the WT (Table 3). The specificity constant was lower in the double mutants for both enzymes than for their respective WTs.

The Arg/Lys270Thr mutations did not significantly perturb the loganic acid–induced spectral changes for either CYP (Fig. 3 and Table 1). Although CYP72A564 Arg270Thr demonstrated comparable steady state kinetic parameters for loganic acid than its WT (Table 2), the corresponding CYP72A565 mutant did not show evidence of loganic acid turnover (Table 3). The Arg/Lys270Thr mutation slightly decreased affinity for loganin (a 1.4-fold increase in *K*_*s*_, Table 1) for both enzymes corresponding with larger Michaelis constants and smaller specificity constants (Tables 2 and 3).

Ser324Glu either reduced affinity for loganic acid (a 2.9-fold increase in *K*_*s*_ for CYP72A564) or eliminated Type I binding (for CYP72A565). The effects of this mutation on loganin binding also differed between the SLASs: CYP72A564 saw a 5.1-fold increase in *K*_*s*_ whereas that of CYP72A565 was unaffected. The effects on steady state kinetics parameters were more uniform—no evidence of loganic acid turnover, larger Michaelis constants, rate constants comparable to the WT, and smaller specificity constants (Tables 2 and 3).

### *In vitro* reconstitution confirms the effects of these mutations on secoiridoid production

**Figure 4:**
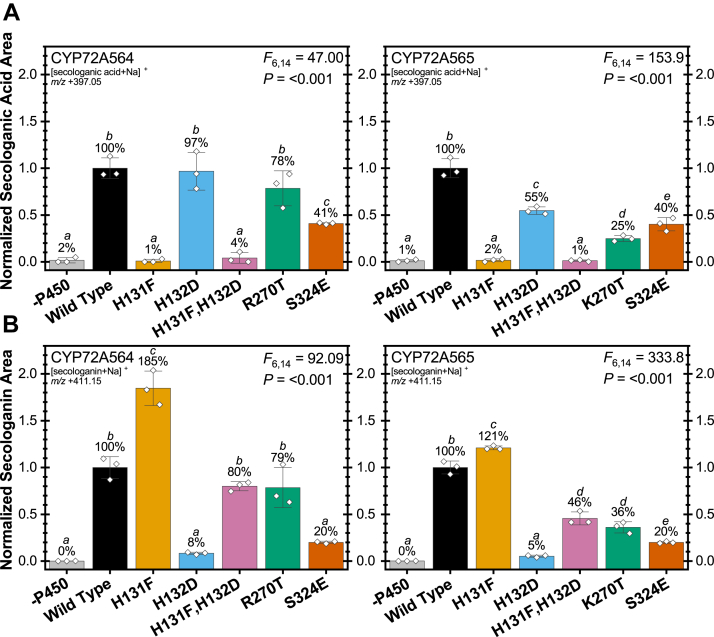
**Secologanic acid, secologanin produced by *Camptotheca*CYP72A564 and CYP72A565mutants.** Triplicate *in vitro* reactions of the listed CYP mutants with *Camptotheca* CPR1 were assayed for secologanic acid produced from loganic acid (*A*) and secologanin produced from loganin (*B*) by LC-MS normalized to an internal standard. The percentages of WT activity (bars ± SD with ◊ denoting individual replicates) were compared by a one-way ANOVA with Tukey’s HSD test used for *post hoc* analysis (α = 0.05). Fig. S7 displays the extracted ion chromatograms used for quantitation. Tables S7–S10 contain the output from the pairwise comparisons.

To evaluate how these mutations affect the production of secologanic acid and secologanin, we reconstituted the CYPs with full-length *Camptotheca* *cytochrome P450 reductase* (CPR)1 as the partner reductase and compared the amount of each secoiridoid produced (Fig. 4). Using the specificity constants from the steady state experiments, we predicted that Arg/Lys270Thr mutants would produce secologanic acid at lower levels, that the Ser324Glu mutants would produce lesser amounts still, and that all other mutants (His131Phe; His132Asp; and His131Phe, His132Asp) would not produce secologanic acid. For assays using loganin, we predicted both His131Phe mutants as the greatest producers of secologanin followed by the WT enzymes, His132Asp and Ser324Glu as the lowest producers of secologanin, and the double mutants as well as Arg/Lys270Thr as lying somewhere in between.

We surprisingly observed that the His132Asp mutant from CYP72A564 produced about the same amount of secologanic acid as its WT protein and that the same mutant from CYP72A565 produced just above half the amount of secologanic acid compared to its WT enzyme (Fig. 4*A*). Each mutant containing His131Phe failed to produce secologanic acid above background levels in accordance with their lack of loganic acid–induced Type I spectra and no increase in NADPH consumption in the presence of this iridoid. For CYP72A564, Arg270Thr only slightly affected secologanic acid production, whereas, for CYP72A565, Lys270Thr substantially decreased secologanic acid production to only one quarter that of the WT. For both enzymes, Ser324Glu lowered activity (as predicted) to approximately 40% the activity of the WT.

In contrast, the pattern of secologanin production was the same for both CYP72A564 and CYP72A565 and roughly followed the pattern predicted by the Type I spectra and specificity constants: His131Phe > WT ≥ His131Phe, His132Asp = Arg/Lys270Thr > Ser324Glu ≥ His132Asp = no P450 (Fig. 4*B*). For CYP72A564, the His131Phe mutant produced almost twice as much secologanin as the WT with the double mutant and Arg270Thr showing no significant change in activity. The His132Asp and Ser324Glu mutations drastically reduced secologanin production. The His131Phe mutation showed a weaker increase in secologanin production with CYP72A565, but the double mutant and Lys270Thr mutation both reduced secologanin production by over half. As with CYP72A564, mutating His132Asp or Ser324Glu in CYP72A565 effectively eliminated secologanin production.

To summarize the effects of mutagenesis on both these P450s, none of the mutations investigated improved secologanic acid production, whereas the His131Phe mutation improved secologanin production for both CYP72A564 and CYP72A565. The adjacent His131 and His132, when mutated independently, toggled selectivity for secologanic acid production from loganic acid (His132Asp) and secologanin production from loganin (His131Phe). Arg/Lys270Thr changes reduced secoiridoid production though not as severely as other mutations. For both CYP72A564 and CYP72A565, Ser324Glu was consistently deleterious to secoiridoid production. Between the His131Phe and His132Asp mutations that toggled selectivity when introduced singularly, double mutants of these *Camptotheca* SLASs made to resemble the other CYP72As sequences annotated as SLS or SLS-like converted loganin to secologanin but not loganic acid to secologanic acid as for the His131Phe single mutants.

### Resurrected SLS, SLAS common ancestor is multifunctional like extant *Camptotheca* enzymes

To probe the concerted effect of these four loci, we expressed and purified the predicted SLS, SLAS common ancestor (Fig. 1*A*; node 34, Figs. S1, S2, and File S1). Because three of the four sites shared residues with the extant *Camptotheca* SLASs (His131, His132, and Arg270), we predicted that this ancestral CYP would be capable of converting loganic acid to secologanic acid *and* loganin to secologanin. We further predicted that this ancestral enzyme would have lower activities than either of the extant *Camptotheca* SLASs owing a shorter evolutionary time to select for secoiridoid synthase activity and Glu324.

**Figure 5:**
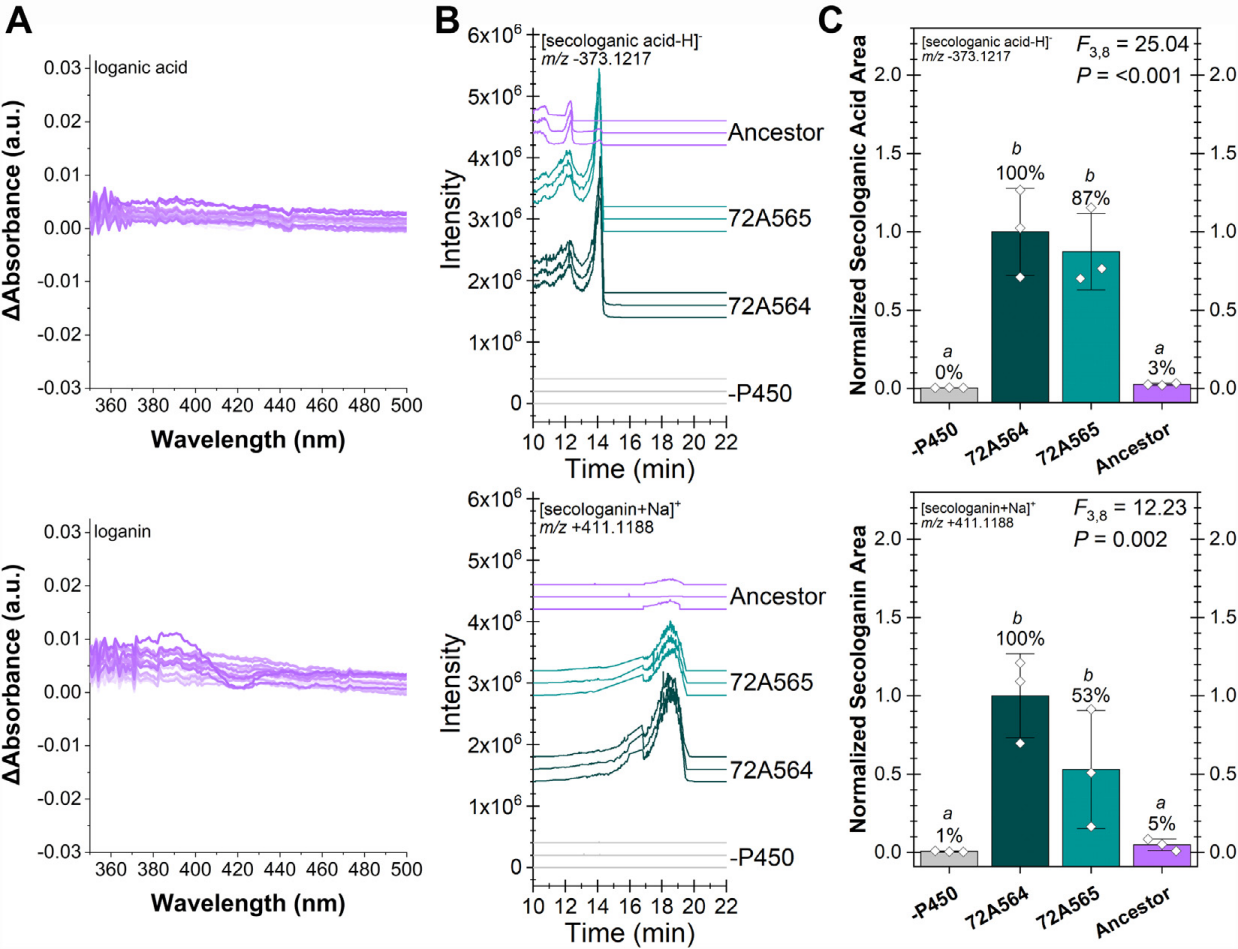
**SLS, SLAS common ancestor Type I binding spectra and secoiridoid production.***A*, difference spectra from 998 nM (*lighter color*) to 1.90 mM (*darker color*) loganic acid (*top*) or loganin (*bottom*) for the SLS, SLAS common ancestor. Fig. S8 and Table 4 record the binding isotherms and parameters, respectively. *B*, extracted ion chromatograms from triplicate *in vitro* reactions of the listed CYPs with *Camptotheca* CPR1 were assayed for secologanic acid (*top*) or secologanin (*bottom*) production by high resolution LC-MS. *C*, the amount of the secoiridoid produced are reported as percentages of CYP72A564 activity (bars ± SD with ◊ denoting individual replicates) and compared by a one-way ANOVA with Tukey’s HSD test used for *post hoc* analysis (α = 0.05). Tables S11 and 12 contain the output from the pairwise comparisons. SLS, secologanin synthase.

After synthesizing and cloning a His_6_-tagged coding sequence into a bacterial expression vector, the SLS, SLAS common ancestor was expressed and purified using the same procedures as for the *Camptotheca* SLASs. As with our SLAS mutants, we first examined whether loganic acid and/or loganin induced Type I spectra (Fig. 5*A*). Up to 1.9 mM loganic acid, no spin shift was observed for this ancestral CYP, whereas loganin yielded perceptible spin shift at concentrations above 0.5 mM. Likewise, the binding isotherms (Fig. S8) and fit parameters (Table 4) show loganin as a weak binder (*K*_*s*_ = 2.3 mM, higher than the highest concentration assayed) and loganic acid as producing no spin shift.

**Table 4:** Type I–binding isotherm parameters of SLS, SLAS common ancestor

Compound	*K*_*s*_*/mM*	ΔA_max_/*mA.U.*
loganic acid	n.d.^a^	n.d.^a^
loganin	2.3 ± 1.0^b^	21.4 ± 6.5^b^

a n.d., no data because one or more fit parameters were not significantly different than zero.

b The apparent *K*_*s*_ is above the highest concentration (1.90 mM) used rendering the nonlinear curve fitting inaccurate.

When reconstituted *in vitro* with full-length *Camptotheca* CPR1, the SLS, SLAS common ancestor did produce secologanic acid from loganic acid *and* secologanin from loganin (Fig. 5*B*)—albeit just above the limit of detection. Indeed, this ancestral CYP showed less than 10% of the activity of either of the extant *Camptotheca* SLASs (Fig. 5*C*). As observed in our previous study (20), CYP72A564 consistently showed higher SLS *and* SLAS activity than CYP72A565.

## Discussion

The ability of *Camptotheca* CYP72A564 and CYP72A565 to both produce and metabolize loganic acid as well as transform loganin into secologanin is unique among known 7DLHs (3, 7, 26) and SLSs (4, 16, 17, 18, 20). By combining ASR with homology modeling, we first implicated four sites as contributing to the multifunctionality of these *Camptotheca* SLASs and then assayed the importance of the identified residues by conducting *in vitro* assays with site-directed mutants. We found that the adjacent residues His131 and His132 in SRS1 are imperative for setting selectivity in these enzymes: His131Phe selects for secologanin production from loganin; His132Asp selects for secologanic acid production from loganic acid. We also found that the effects of modifying Arg/Lys270 along the predicted substrate entry channel in SRS3 moderately reduce the SLAS and SLS activities of these P450s. The effects of modifying Ser324 at the start of the I-helix in SRS4 severely reduces both activities. The resurrected SLS, SLAS common ancestor (with His131, His132, Arg270, and Glu324) demonstrated both SLAS and SLS activity—albeit less readily than CYP72A564 and CYP72A565, the extant *Camptotheca* SLASs.

That the His132Asp mutant did not undermine secologanic acid production is puzzling given the absence of substrate-induced Type I spectra or increased NADPH consumption. A lack of Type I spectra is not unknown for compounds that bind tightly to P450s (27), presenting the possibility that loganic acid binds without strongly perturbing the heme electronic environment. The proximity of His132 to the docked loganic acid in the CYP72A564 and CYP72A565 homology models (20) (File S2) suggests an adjacent positioning of Asp132 and the carboxylic acid moiety of the substrate. Coordination of a cation (*e.g.*, sodium or potassium) by the protein and substrate carboxylic acids would transform unfavorable Coulombic interactions into stable binding interactions. In fact, the lack of spin shift indicated by the Type I spectra furnish a compelling explanation for NADPH consumption rates near background levels. The shift from low-spin (S = 1/2) to high-spin (S = 5/2) heme iron induced by removal of water as the sixth ligand increases the iron’s reduction potential (28). This increase in potential brings it nearer to that of NADPH and CPR, thus improving the electron transfer to the heme (29, 30). Without this spin shift (*i.e.*, as was the case for the His132Asp mutants in the presence of loganic acid), reduction to iron(II) is greatly slowed. In this case, the rate of electron transfer to the P450 for turnover was sufficiently slow to be masked by the background rate of NADPH consumption by CPR due to uncoupling.

The inability of these His132Asp mutants to produce much secologanin further suggests that charges are not the only biochemical characteristic at play in loganic acid and loganin binding. Besides the exchange of a positively charged side chain for a negatively charged one, the His132Asp change causes the loss of an H-bond donor. This loss of an H-bond donor disrupts loganin binding and, thereby, turnover. The reverse selectivity for loganin observed for His131Phe likely involves perturbed interactions with the carboxylate of loganic acid too. Replacing a likely cationic, H-bond donor (His) with a similarly sized hydrophobic residue (Phe) disfavors the binding of anions (like loganic acid) while improving affinity for more hydrophobic functional groups like esters (like loganin).

Perturbing interactions with the carboxylic acid moiety of loganic acid and loganin were not the lone determinants of modifying enzymatic activities. The inactivity of both Ser324Glu mutants corroborates the importance of this hinging region in the I-helix for active site opening and closing in as described for bacterial CYPs (24, 25). Like bacterial CYP MycG (25), the homology model of CYP72A565 has an H-bonding network including the I-helix (Ser324) and G-helix (Ser274); contrastingly, nearly all members of the SLS lineage have Arg274. This conserved Arg274 residue may work in concert with the Glu324 as Ser274 and Ser324 do in the SLASs countering our observations of reduced activities for the Ser324Glu SLAS mutants. Consistent with this hypothesis, the SLS, SLAS common ancestor (with a mismatched Ser274 and Glu324) had poorer activity than the *Camptotheca* SLASs (with Ser274 and Ser324).

Based on our results, any attempts at further improving secologanic acid production by these *Camptotheca* SLASs should target His132 building from the Asp mutant. Most mutations one might suggest, however, present possible drawbacks: increasing the chain length (Glu) might push loganic acid out of position for catalysis within the enzyme active site; replacement with another H-bond donor might be too long (Arg, Gln, Lys) or too short (Ser, Thr). The modest changes in size while retaining an H-bond donor presented by mutation to Asn or Tyr render them the best options for rational design at this site. Our results discourage attempts to mutate His132 to hydrophobic residues where secologanic acid production from loganic acid is desired because His132Phe eliminated this activity. Where increased secologanin production and/or selectivity for loganin are desired, the increase in SLS activity related to the His131Phe mutation supports attempting comparable shifts to nonpolar sidechains at His132. The less severe effects observed when modifying Arg/Lys270 support this region as another area worth exploring for modifying these enzymes’ activities.

The comparatively efficient SLS activity of *Camptotheca* CYP72A564 and CYP72A565 render them reasonable candidates for heterologous production of TIAs (*e.g.*, ibogamine, ajmaline, reserpine, vinblastine; Fig. 1). The multifunctionality of these two *Camptotheca* SLASs, however, presents the likely accumulation of another metabolite—secologanic acid—disruptive of strictosidine production. The production of secologanic acid is especially problematic because there are mixed reports (12, 18, 31) as to whether loganic acid *O*-methyltransferases (LAMTs) can efficiently produce secologanin from secologanic acid. In the event that it is not quickly converted to secologanin, secologanic acid becomes coupled to tryptamine (enzymatically or nonenzymatically) *via* a Pictet-Spangler condensation (32). The strictosidinic acid produced by this reaction then spontaneously lactamizes to yield strictosamide *in lieu* of strictosidine. In the camptothecin-producing organisms *Camptotheca* (33) and *Ophiorrhiza* (18), strictosamide feeds the species-specific pathway producing camptothecin; in other TIA biosynthetic pathways such as those found in *Catharanthus* and *Rauvolfia* (10, 34, 35, 36), producing strictosamide problematically diverts carbon and nitrogen from the production of desired TIAs like vinblastine and ajmaline.

The simultaneous increase in secologanin production and loss of activity toward loganic acid present the His131Phe mutants of *Camptotheca* CYP72A564 and CYP72A565 as excellent SLS candidates for heterologous production of strictosidine and, thereby, several frequently targeted TIAs. Indeed, these *Camptotheca* enzymes are quality alternatives to the *Catharanthus* isoforms employed to produce strictosidine in *S. cerevisiae* (15) where their use led to an undesirable accumulation of loganin. In our hands, the *Catharanthus* SLS has poorer expression in *E. coli* and stability than these *Camptotheca* CYPs (20). This poorer *ex vivo* stability suggests that *Catharanthus* CYP72A1 and its multiple isoforms are generally less stable, explaining the intractability of loganin accumulation when several of these isoforms were coexpressed or overexpressed in yeast (15). The amenability of *Camptotheca* CYP72A564 and CYP72A565 to heterologous expression in *E. coli* and purification to considerable concentrations (up to ∼50 μM) mark these enzymes as preferable to *Catharanthus* CYP72A1 when employing heterologous production systems.

Secologanic acid production as facilitated by the SLASs of *Camptotheca* appears unique among TIA- and camptothecin-producing plants (17, 18, 33). The nearly identical SRSs of *Camptotheca* CYP72A564, CYP72A565 and the SLS, SLAS common ancestor support the multifunctionality of the *Camptotheca* enzymes as the ancestral state of the last common ancestor of TIA-producing plants. The presence of a nonfunctional LAMT homolog in *Camptotheca* (37) suggests that this ancestral TIA-producer employed loganin, secologanin, and strictosidine as intermediates (perhaps along with secologanic acid, strictosidinic acid, and strictosamide also present). Continuing with this hypothesis, the lineage leading to *Camptotheca* subsequently lost the ability to produce loganin, and the resulting biosynthetic pathway evolved to produce camptothecin *via* strictosamide derived solely from secologanic acid (33).

Contrasting this route to TIAs through secologanic acid and strictosidinic acid in the *Camptotheca* lineage, the SLS ancestor of the core asterids lost the ability to utilize loganic acid with the His131Phe mutation and, thereby, became specialized for the conversion of loganin into secologanin. For the extant, multifunctional *Camptotheca* CYPs, the His131Phe mutation alone increased loganin activity while eliminating loganic acid activity, and the His132Asp mutation decimated loganin activity. The His132Asp mutation therefore likely occurred first. Selective pressure subsequently caused the SLS-rescuing His131Phe mutation to maintain sufficient levels of secologanin for TIA production. The lineage leading to *Camptotheca* at some point in time substituted Ser324 in place of the ancestral Glu present in the SLSs of the core asterids. The Lys270Thr replacement likely occurred after the differentiation of the lamiids and campanulids as both campanulid SLSs (*Lonicera* and *Nothapodytes*) retain the ancestral Lys.

The high sequence homology, shared motifs within clades at the four loci identified in this report, and common biosynthetic intermediates (*i.e.*, loganin, secologanin, and strictosidine) of TIA-producing plants strongly suggest an evolutionarily shared origin of TIA biosynthesis. Although Rai *et al.* (38) rightly conclude that an ancient origin for TIA biosynthesis requires a repeated loss of such capabilities across plant species, the function of secologanin production *and* the shared development of CYP72As for this function when taken together are in greater agreement with the loss of TIA-production in multiple lineages than convergent evolution of these metabolites. Furthermore, the apparent loss of LAMT activity in the lineage leading to *Camptotheca* (37) and the differentiation of lamiid SLSs by Thr270 from those of other asterids bolster the claim of an ancient TIA origin up to secologanin and strictosidine production.

Contrastingly, camptothecin production in *Camptotheca*, *Nothapodytes*, and *Ophiorrhiza* appears to have arisen by convergent evolution. The divergence of *Camptotheca* in its biosynthetic intermediates after loganic acid (18, 33) and the occurrence of camptothecin production in three distinct lineages (Cornales, *Camptotheca*; Aquifoliales, *Nothapodytes*; *Ophiorrhiza*, Gentianales) substantiate convergent evolution. The differential trajectories of whole-genome duplications and development of camptothecin biosynthetic genes observed in *Camptotheca* and *Ophiorriza* (37, 38) further strengthens this claim. Comparable chromosome-level genome assemblies and analyses for *Nothapodytes* can illuminate this matter more; however, continued elucidation of camptothecin biosynthesis in these plants alongside comparison of the enzymes employed will clarify the extensiveness of these instances of convergent evolution.

Although the use of ASR to identify key developments in CYP specialization is well reported for animal CYPs (39, 40, 41, 42), our work highlights the usefulness of ASR and related methodologies for investigating CYP specialization in plant biosynthetic pathways. In light of the accumulating knowledge of plant specialized metabolism, the large number of CYPs in these pathways, and the burgeoning collections of genomic resources, ASR is emerging as a powerful tool to investigate how particular enzymes and the biosynthetic pathways they constitute have evolved through time. Increasing such studies provides guidance for the redesign of enzymes and creation of new-to-nature biosynthetic pathways needed to achieve greener chemical production *via* synthetic biology.

Each of the four sites identified by our combined ASR and homology modeling approach demonstrated important effects in differentiating SLAS from SLS activity. None of the mutations increased SLAS activity to any extent, whereas the effects on SLS activity were more variable. Intriguingly for improving heterologous production of strictosidine and thereby TIAs, mutating His131 to Phe in either of the *Camptotheca* SLASs simultaneously increased secologanin production *in vitro* and eliminated detectable loganic acid binding or turnover. Conversely, mutating His132 to Asp reduced secologanin production to background levels while the ability to produce secologanic acid was retained. As genetic and biochemical knowledge of specialized metabolism in plants continues to increase, studying the evolutionary development of biosynthetic pathways using methods like ASR will aid the design of these pathways and the enzymes that constitute them.

## Experimental procedures

### General

PCR reagents, plasmid purification, restriction enzymes, and T4 DNA ligase were used as recommended by New England Biolabs (NEB) unless otherwise noted. Vectors used in this study included the bacterial expression vectors pCWori (43) and pET28a (Novagen). *E. coli* cell strains used in this study included Top10 (Invitrogen), DH5α, and BL21(DE3) (NEB). All reagents were used as received from commercial sources. Loganin (Cayman Chemical) and loganic acid (Arctom Chemicals) were 98+% pure.

LC-MS analyses of the *Camptotheca* SLAS mutants were conducted using a Shimadzu LC-MS2010EV system at the Institute for Genomic Biology, University of Illinois Urbana-Champaign. High resolution LC-MS analyses of the WT *Camptotheca* SLASs and the SLS, SLAS common ancestor were performed by Dr Alexander Ulanov in the Metabolomics Laboratory of the Roy. J. Carver Biotechnology Center at the University of Illinois Urbana-Champaign using a Dionex Ultimate 3000 series HPLC system (Thermo Scientific) with Q-Exactive MS system (Thermo Scientific). Spectroscopic measurements were recorded with a Cary UV-Vis Bio100 dual beam spectrophotometer or Molecular Devices SpectraMax M-series plate reader as appropriate.

### Ancestral sequence reconstruction

CYP72A sequences annotated as secologanin synthases (SLSs; 161 hits), 7-DLHs (23 hits), SLS-like (30 hits), or 7DLH-like (0 hits) in GenBank were selected for the ASR. After curation to remove duplicates and CYP72A genes from species not known to produce loganic acid, the sequences chosen for inclusion in our analysis totaled 21 CYP72A genes, including three from *Camptotheca* (Table S1). *Arabidopsis thaliana* CYP734A1, a member of the closest subfamily to CYP72A, was chosen to root the tree as was done for a phylogeny of the whole CYP72A subfamily (21). The sequences were aligned with MEGA X (44) using MUSCLE (45) with the default parameters.

The model analysis tool of MEGA X identified the Le-Gascuel model (46) with a discrete gamma distribution (LG+G) as the best substitution model to use for the maximum likelihood phylogenetic tree. The initial tree was obtained by BioNJ; tree topology, branch lengths, and rate parameters were optimized in a bootstrap analysis with 1000 replicates. The results from this bootstrap analysis served as the initial tree used to infer the ancestral states of nodes in the phylogeny using the LG+G model treating the rates among sites as a gamma distribution with four gamma categories. The node identifications (Fig. S1), a multiple sequence alignment featuring all extant and ancestral sequences (File S1), and the amino acid probabilities for all ancestral sequences (File S1) are appended as supporting materials.

### Homology modeling

The three-dimensional protein models based on single-template homology are the same as previously reported (20, 47) and were constructed utilizing MOE (Chemical Computing Group Inc.). As previously detailed, the template structure was determined *via* a multiple sequence alignment in MOE with all substrate-free CYPs in the PDB as of January 2018. All CYPs reported herein were modeled on substrate-free CYP2D6 (PDB 2F9Q) (48). Loganic acid was docked into each CYP72A before final minimization. The final models with docked loganic acid are supplied as ∗.pdb files (File S2).

### Site directed mutagenesis

We employed a three-step PCR method to create full-length, C-terminally His_6_-tagged mutant ORFs for expression using the primers given in Table S2. CYP72A564 (GenBank MN815881) and CYP72A565 (GenBank MN815882) cloned into pCW from our previous study (20) were used as templates for mutagenesis. All PCR reactions were performed with Q5 High Fidelity DNA Polymerase (NEB) according to the manufacturer’s recommendations with the annealing temperature for the primary and tertiary PCR reactions set at 69 °C. DpnI (20 U; NEB) digestions in Q5 reaction buffer lasted at least 3 h at 37 °C before heat inactivation at 80 °C for 20 min. Buffer and excess reagents were removed from the primary and tertiary PCR products using a GeneJET PCR Purification Kit (Thermo Fisher Scientific) according to the manufacturer’s recommendations.

For the first PCR step, the 5′ block was produced using a 5′ NdeI primer at the N-terminus of the CYP and a mutant internal primer; the 3′ block was produced with a 3′ His_6_ XbaI primer corresponding to the C-terminus of the CYP with another mutant internal primer (Table S2). The mutant primers were designed to contain an overlap of at least four nucleotides for later generation of full-length inserts. Following cycles with a 69 °C annealing temperature, excess reagents were removed from these 5′ and 3′ blocks using a PCR purification kit.

For the second PCR step, which created a pool of full-length, dsDNAs, approximately equimolar amounts of the 5′ and 3′ products were combined and amplified by PCR for 20 cycles with a low (40 °C) annealing temperature. DpnI digestion preceded a third PCR seeded from the second to eliminate contaminating WT plasmid. This third PCR used the gene-specific 5′ and 3′ primers, 30 cycles, and a 69 °C annealing temperature to amplify enough of the full-length mutant transcript for subsequent insertion into the pCW expression vector.

Tertiary PCR products were digested with NdeI (20 U; NEB) and XbaI (20 U; NEB) at 37 °C in 1× CutSmart buffer (NEB) for at least 2 h. Where a partial digest was required, only 2 U of enzyme were added, and the enzymes were heat denatured for 20 min at 80 °C after the final 15 min of the 37 °C digestion. Restriction endonuclease–digested PCR products were purified from low-melt agarose (Research Products International) using β-agarase (NEB) digestion and isopropyl alcohol precipitation. Purified inserts were ligated into pCW using T4 DNA ligase (NEB). Dideoxy-sequencing over the full length of each coding sequence confirmed mutagenesis at only the desired position(s).

### SLS, SLAS ancestor gene construction

A codon-optimized, His_6_-tagged gene encoding the predicted protein sequence was synthesized by Integrated DNA Technologies. PCR amplification of this gene with NdeI- and XbaI-containing primers, restriction digestion, purification, and ligation into pCW were performed as described for the mutants.

### CYP, CPR protein expression and purification

Mutant *Camptotheca* CYPs were purified as previously reported for the WT enzymes (20), except Mg^2+^ and ATP were omitted from the resuspension step. Typical yields from 2 l of culture were 1 to 2 ml of 10 to 60 μM P450.

Full-length *Camptotheca* CPR1 was also purified as previously reported (20) except for the replacement of Tris-HCl–containing buffers with sodium-Mops (pH 7.3) buffers containing 10% glycerol (49).

### CYP, CPR characterization

The concentration of properly folded CYPs (the P450 fraction) in each preparation was determined using an extinction coefficient of 106,000 M^-1^ cm^-1^ at 450 nm in a CO-reduced minus CO-oxidized difference spectrum (50). CPR activities (in mU/μl) were determined using the cytochrome *c* reduction method of Guengerich *et al.* (50).

### Substrate-induced spectral shift assays (type I binding)

Concentrated CYPs were diluted to 0.60 μM in 100 mM NaPO_4_ (pH 7.5) and their spectra obtained from 300 to 700 nm against a reference cuvette containing only buffer. Successive aliquots of loganic acid, loganin, or buffer were added to both the sample and reference cuvettes, respectively, to account for absorbance not associated with the CYPs. To correct for variations occurring from the removal and repositioning of cuvettes, spectra were first internally normalized by subtracting the absorbance at 700 nm from all other wavelengths. Then, the difference spectra were obtained by subtracting the appropriate buffer control spectrum from the substrate-containing spectrum of the same sample volume.

Binding isotherms from these spectra plotted the difference in ΔAbs from peak (at ∼389 nm) to trough (at ∼419 nm) against substrate concentration. The binding constants are reported with standard error from the curve fitting regression conducted in OriginPro2019 to a one-site binding model: ΔAbs = (*ΔA*_*max*_ ∗ [S])/(*K*_*s*_ + [S]) where *ΔA*_*max*_ is the maximum difference between peak and trough (proportional to the amount of CYP bound to the substrate), [S] is the substrate concentration, and *K*_*s*_ is the binding constant. 95% confidence intervals for each parameter were calculated using an *F*-test (Tables S3 and S4). Any fits where at least one parameter’s confidence interval included zero were interpreted as representing no binding of the compound assayed.

### Steady state kinetics

To estimate the rate of product formation, the rate of NADPH consumption was monitored spectroscopically in polystyrene 96-well microtiter plates with each 100 μl reaction (0.211 cm pathlength) performed at 30 °C with technical triplicates. Individual wells contained 0.5 mU/μl full-length *Camptotheca* CPR1, 25 pmol P450 (250. nM), 60 μM 1,2-dilauroyl-sn-glycero-3-phosphocholine (DLPC) (final concentration of 0.6% v/v methanol), no substrate or 50.0 μM to 5.00 mM substrate in 100 mM NaPO_4_ (pH 7.5). A master mix containing the CPR, P450, and DLPC was preincubated on ice for 2 h before adding substrate within the microtiter plate wells. Reactions were initiated by the addition of NADPH (∼0.8 mM final concentration), shaking for 15 s before reading and monitoring at 340 nm every 15 s with shaking for 3 s between reads.

Initial rates were estimated *via* a linear regression of the absorbance *versus* time data, converted from a.u. min^−1^ to μM NADPH min^−1^ using an extinction coefficient of 6220 M^−1^ cm^−1^. Subtracting the rate of consumption in the absence of substrate from substrate-containing assays corrected for the background rate of NADPH turnover by CPR. Plotting these corrected rates against substrate concentration in OriginPro2019 and fitting to the Michaelis–Menten equation by nonlinear curve fitting yielded estimates of the kinetic parameters (Fig. S6). As with the substrate-binding isotherms, 95% confidence intervals for each parameter were calculated by an *F*-test (Tables S5 and S6), and we interpreted any fits where at least one parameter’s confidence interval included zero as representing no fit (and nonsignificant turnover) for the compound assayed.

### *In vitro* reconstitutions

SLAS activity was reconstituted *in vitro* with full-length *Camptotheca* CPR1 in the presence of DLPC (20). Fifty microliter reactions in 100 mM NaPO_4_ (pH 7.5) buffer were started by the addition of a master mix to 12.5 pmol P450 (250. nM final concentration) or buffer. In the final reaction volume, the master mix contained 60 μM DLPC (0.6% v/v methanol final concentration), 0.5 mU/μl CPR, 10 mU/μl glucose-6-phosphate dehydrogenase, 250 μM substrate, 500 μM NADPH, and 1 mM glucose-6-phosphate. The master mix was created by adding the appropriate volume of a DLPC stock dissolved in methanol to the phosphate buffer before vigorous mixing. The enzymes and substrates were then added, mixed gently, and added to the P450 aliquots to commence the reaction. Upon addition of the master mix, reactions were incubated at 30 °C for 120 min before quenching with 50 μl methanol (for reactions with loganin) or acetonitrile (ACN) (for reactions with loganic acid) containing 250 μM caffeine as an internal standard. Secoiridoid production was then assessed for each mutant and WT enzyme from triplicate assays *via* LC-MS.

### Liquid chromatography-mass spectrometry

A Shimadzu LCMS-2010EV system was used to analyze *in vitro* reactions. A Phenomenex Gemini C_18_ 110 Å (5 μM × 150 mm × 2 mm) column maintained at 40 °C separated analytes from a 10 μl injection using a gradient at a 0.200 ml/min flow rate. The gradient for assays containing loganin was 10% ACN in 0.2% (v/v) acetic acid/water, 0 min; linear ramp to 30% ACN at 25 min; linear ramp to 98% ACN at 26 min; linear ramp to 10% ACN at 34 min; hold at 10% ACN until 35 min. The gradient for assays containing loganic acid was 0 min, 2% ACN; linear ramp to 15% ACN at 15 min; linear ramp to 60% ACN at 22.5 min; linear ramp to 98% ACN at 25 min; hold at 98% ACN until 27.5 min; linear ramp to 2% ACN at 27.6 min; hold at 2% ACN until 32.5 min. A photodiode array detector maintained at 40 °C with 1.2 nm slit width monitored 190 to 800 nm at 1.5625 Hz.

Selective ion monitoring for caffeine ([M + H]^+^ 195.05), loganin ([M+Na]^+^ 413.15), and secologanin ([M+Na]^+^ 411.15) after electrospray ionization was used to quantitate these compounds in assays starting with loganin. Parameters for the mass spectrometer were optimized using the instruments AutoTune function: Nebulizing gas flow, 1.5 l/min; heat block temperature, 200 °C; interface temperature, 250 °C; interface bias voltage, +4.5 kV; interface current, 1.40 μA; CDL temperature, 230 °C; CDL voltage, −20.0 V; focus lens, −2.5 V; entrance lens, −45.0 V; RF gain, 4335; RF offset, 5145; prerod bias, −3.6 V; main-rod bias, −3.5 V; aperture voltage, +0.0 V; conversion dynode, −8.0 kV; detector voltage, −2.10 kV.

Selective ion monitoring for caffeine ([M + H]^+^ 195.10), loganic acid ([M+Na]^+^ 399.05), and secologanic acid ([M+Na]^+^ 397.05) after electrospray ionization was used to quantitate these compounds in assays starting with loganic acid. Parameters for the mass spectrometer were optimized using the instruments AutoTune function and the same as for the loganin assays.

The secoiridoids and the internal standard (caffeine) were quantitated from their extracted ion chromatograms using LabSolutions software (Shimadzu). The secoiridoid signal was divided by the caffeine signal, the WT reactions averaged, and each individual reaction divided by this value to yield the normalized secoiridoid production as a percentage of WT activity. A one-way ANOVA with Tukey’s HSD test for *post hoc* pairwise comparison (α = 0.05) compared the activity of the mutants (Tables S7 and S8).

High resolution LC-MS of samples were analyzed using the Q-Exactive MS system in the Metabolomics Laboratory of Roy J. Carver Biotechnology Center, University of Illinois at Urbana-Champaign. The software Xcalibur 3.0.63 was used for data acquisition and analysis. The Dionex Ultimate 3000 series HPLC system used includes a degasser, an autosampler, a diode array detector, and a binary pump. The LC separation was performed using the same column, solvent system, and program as for loganic acid reported above. The autosampler was set to 10 °C with the injection volume of 10 μl. Mass spectra were acquired under both positive electrospray ionization (spray voltage: 3.5 kV) and negative electrospray ionization (spray voltage: −2.5 kV): auxiliary gas flow rate, 65; aux gas flow rate: 20; sweep gas flow rate, 4; capillary temp, 300 °C; auxiliary gas heater temp, 500 °C. The resolution was set to 70,000. The AGC target was 1 × 10^6^ with a maximum injection time of 200 ms. The scan range was *m/z* 50 to *m/z* 750. The integrated areas for the secoiridoids secologanin ([M+Na]^+^ 411.1188) and secologanic acid ([M-H]^−^ 373.1217) as well as caffeine ([M + H]^+^ 195.0876) were analyzed as reported above.

## Data availability

All data are presented in the article and its supporting information.

## Supporting information

This article includes supporting information: Supporting Information (Tables S1–S12; Figs. S1–S8), Supporting Files S1 (Full multiple sequence alignment with all ancestral sequences; Ancestral sequences site-by-site amino acid probabilities), and S2 (Homology model pdb files).

## Conflict of interest

The authors declare that they have no conflicts of interest with the contents of this article.
