# Supplementary material for: Single mutations toggle the substrate selectivity of multifunctional Camptotheca secologanic acid synthases
Source: J Biol Chem. 2022 Jul 6;298(9):102237. doi: 10.1016/j.jbc.2022.102237 (PMC9424959; doi:10.1016/j.jbc.2022.102237)
Supplement: Supporting Information [file mmc3.pdf]

Supporting Information

Single mutations toggle the substrate selectivity of multifunctional *Camptotheca* secologanic acid synthases (CYP72As)

Justin C. Miller[1], Mary A. Schuler[2,3,4]*

[1]Department of Chemistry, University of Illinois Urbana-Champaign, Urbana, Illinois, USA 61801.
[2]Department of Cell and Developmental Biology, University of Illinois Urbana-Champaign, Urbana, Illinois, USA 61801.
[3]Department of Biochemistry, University of Illinois Urbana-Champaign, Urbana, Illinois, USA 61801.
[3]Department of Plant Biology, University of Illinois Urbana-Champaign, Urbana, Illinois, USA 61801
*Author to whom correspondence should be addressed (maryschu@illinois.edu)

Tables S1-S12
Figures S1-S8

Table S1. GenBank accession numbers of sequences used for ancestral sequence reconstruction.

GenBank Accession ID	Species	Identifiers and Notes	Label Used in Phylogenetic Trees
MN815881	Camptotheca acuminata	Secologanic acid synthase; CYP72A564	Caa SLAS CYP72A564 | MN815881
MN815882	Camptotheca acuminata	Secologanic acid synthase; CYP72A565	Caa SLAS CYP72A565 | MN815882
MN815883	Camptotheca acuminata	Secologanic acid synthase-like; CYP72A730	Caa CYP72A730 | MN815883
Q05047.1	Catharanthus roseus	Secologanin synthase; CYP72A1	Cra SLS CYP72A1 | Q05047.1
AGX93062.1	Catharanthus roseus	7-deoxyloganic acid hydroxylase; CYP72A224	Cra 7DLH | AGX93062.1
AGX93046.1	Amsonia hubrichtii	Secologanin synthase-like protein	Ahu SLS | AGX93046.1
AGX93058.1	Amsonia hubrichtii	7-deoxyloganic acid hydroxylase-like protein	Ahu 7DLH | AGX93058.1
AGX93045.1	Cinchona calisaya	Secologanin synthase-like protein	Cca SLS | AGX93045.1
AGX93057.1	Cinchona calisaya	7-deoxyloganic acid hydroxylase-like protein	Cca 7DLH | AGX93057.1
AGX93044.1	Lonicera japonica	Secologanin synthase-like protein	Lja SLS | AGX93044.1
AGX93056.1	Lonicera japonica	7-deoxyloganic acid hydroxylase-like protein	Lja 7DLH | AGX93056.1
AQW38832.1	Nothapodytes nimmoniana	Secologanin synthase-like protein	Nni SLS | AQW38832.1
ATN39844.1	Olea europaea	7-deoxylaganic acid hydroxylase-like protein	Oeu 7DLH | ATN39844.1
BAP90521.1	Ophiorrhiza pumila	Secologanin synthase	Opu SLS | BAP90521.1
AGX93047.1	Rauvolfia serpentina	Secologanin synthase-like protein	Rsa SLS | AGX93047.1
AGX93059.1	Rauvolfia serpentina	7-deoxylaganic acid hydroxylase-like protein	Rsa 7DLH | AGX93059.1
BBI55002.1	Swertia japonica	Secologanin synthase	Sja SLS | BBI55002.1
AGX93048.1	Tabernaemontana elegans	Secologanin synthase-like protein	Tel SLS | AGX93048.1
AGX93060.1	Tabernaemontana elegans	7-deoxylaganic acid hydroxylase-like protein	Tel 7DLH | AGX93060.1
AGX93049.1	Vinca minor	Secologanin synthase-like protein	Vmi SLS | AGX93049.1
AGX93061.1	Vinca minor	7-deoxylaganic acid hydroxylase-like protein	Vmi 7DLH | AGX93061.1
NP_180239.1	Arabidopsis thaliana	CYP734A1; outgroup used to root the tree	At CYP734A1 | NP_180239.1

Table S2. Primers used in this study.

Name	Sequence (5'→3')
Caa SLAS NdeI 5'	ATTACATATGGAAATTCAAATGGACGTTCTGTAC
CYP72A564 His_6 XbaI 3'	TAATTCTAGATCAGTGGTGGTGGTGGTGGTGACATTTGAGCCTGC GGAAGATC
CYP72A564 H131F fwd	TAACAACTTTCAAAAGAATtttCATGCATACAGTCCGTTGACCAAG
CYP72A564 H132D fwd	TAACAACTTTCAAAAGAATCACgATGCATACAGTCCGTTGACCAA G
CYP72A564 H131F,H132D fwd	CAACCATAACAACTTTCAAAAGAATtttgATGCATACAGTCCG TTGACCAAG
CYP72A564 SRS1 rev	GTTATGGTTGGCCAAGATATCCTTG
CYP72A564 R270T fwd	AATCaccGAGAGGTTGTCAGGTATCATCAACAG
CYP72A564 R270T rev	GATTTCTCTGTCAACTTCCCTCATCC
CYP72A564 S324E fwd	CATCgaaGAATGCAAGCTATTCTACTTCG
CYP72A564 S324E rev	GATGACATCCTCAATACTCATTCCAGC
CYP72A565 His_6 XbaI 3'	TAATTCTAGATCAGTGGTGGTGGTGGTGGTGACACTTGATCTTGC GGAAGACCAC
CYP72A565 H131F fwd	CAACGACTTCATGAAGAATtttCATGCATACAACCCACTAACCAAA TTC
CYP72A565 H132D fwd	GAATCACgATGCATACAACCCACTAACCAAATTC
CYP72A565 H131F,H132D fwd	CAACCACAACGACTTCATGAAGAATtttgATGCATACAACCC ACTAACCAAATTC
CYP72A565 SRS1 rev	GTTGTGGTTGGCCAATATATCC
CYP72A565 K270T fwd	GATAaccGACAGGTTGTCGGGGATCATC
CYP72A565 K270T rev	TATCTCTCCATCAACTTCCCTCATCC
CYP72A565 S324E fwd	CATCgaaGAATGCAAGCTTTTCTACTTTGCG
CYP72A565 S324E rev	GATGACATCTTCAATACTCATGCCAGC
SLS, SLAS Ancestor fwd	ATTACATATGGAGATGGATTTACTTTACAAGTCCATTGCC
SLS, SLAS Ancestor rev	ATTATCTAGATCAGTGGTGGTGATGATGGTGGCTC

Lowercase text denotes nucleotide mutation. Highlighted text denotes mutated codon.

Table S3. 95% Confidence Intervals of CYP72A564 Substrate Binding Isotherms.

Substrate	Mutation	K_s / *mM*			ΔA_{max} / *mA.U.*		
		Lower	Upper	Group	Lower	Upper	Group
loganic acid	Wild Type	0.763	1.003	a	36.8	42.4	a
	H131F	--	0.002		1.5	2.2	
	H132D	--	--		--	--	
	H131F,H132D	--	0.579		0.1	0.8	
	K270T	0.916	2.561	a,b	30.9	57.9	a
	S324E	1.157	8.284	b	9.8	40.5	a
loganin	Wild Type	0.218	0.302	a	44.0	49.4	a
	H131F	0.048	0.062	b	60.8	64.2	b
	H132D	1.097	1.302	c	40.7	44.9	a
	H131F,H132D	0.232	0.286	a	62.5	67.3	b
	K270T	0.354	0.389	d	45.4	47.2	a
	S324E	0.767	2.421	c	22.7	44.9	a

Table S4. 95% Confidence Intervals of CYP72A565 Substrate Binding Isotherms.

Substrate	Mutation	K_s / *mM*			ΔA_{max} / *mA.U.*		
		Lower	Upper	Group	Lower	Upper	Group
loganic acid	Wild Type	1.77	3.47	a	35.7	56.8	a
	H131F	--	0.01		0.4	0.8	
	H132D	--	0.98		--	4.3	
	H131F,H132D	--	0.02		0.7	1.4	
	K270T	1.06	4.65	a	15.2	40.8	a
	S324E	--	--		--	--	
loganin	Wild Type	0.700	1.016	a	46.5	56.2	a,b
	H131F	0.256	0.312	b	56.1	60.3	a
	H132D	1.596	10.707	c	14.2	60.6	a,b,c,d
	H131F,H132D	0.264	0.453	b	38.4	47.3	b
	K270T	1.039	1.395	d	31.1	36.8	c
	S324E	0.540	1.864	a,c,d	13.1	25.5	d

Table S5. 95% Confidence Intervals of CYP72A564 Steady State Kinetic Parameters.

Substrate	Mutation	K_M / *mM*			V_{max} / μM min^{-1}		
		Lower	Upper	Group	Lower	Upper	Group
loganic acid	Wild Type	1.53	5.20	a	2.65	5.00	a
	H131F	--	--		--	--	
	H132D	--	--		--	--	
	H131F,H132D	--	--		--	--	
	R270T	2.64	4.91	a	4.28	6.01	a
	S324E	--	--		--	--	
loganin	Wild Type	0.197	0.296	a	2.84	3.16	a
	H131F	0.041	0.068	b	1.89	2.06	b
	H132D	1.317	1.582	c	3.28	3.55	c
	H131F,H132D	0.131	0.922	a,d	1.21	2.13	b
	R270T	0.519	2.017	c,d	3.84	6.55	d
	S324E	1.063	1.485	c	2.34	2.69	e

Table S6. 95% Confidence Intervals of CYP72A565 Steady State Kinetic Parameters.

Substrate	Mutation	K_M / *mM*			V_{max} / μM min^{-1}		
		Lower	Upper	Group	Lower	Upper	Group
loganic acid	Wild Type	2.80	4.64	a	4.27	5.63	a
	H131F	--	--		--	--	
	H132D	--	--		--	--	
	H131F,H132D	--	--		--	--	
	K270T	--	--		--	--	
	S324E	--	--		--	--	
loganin	Wild Type	0.578	0.865	a	3.54	4.09	a
	H131F	0.119	0.457	a	1.61	2.24	b
	H132D	0.966	9.687	b,c	2.58	7.72	a,c,d
	H131F,H132D	1.501	1.920	b	6.78	7.57	c
	K270T	0.425	3.526	a,b,c	1.65	3.52	b,d
	S324E	2.155	5.006	c	3.79	5.90	a

Table S7. Tukey's HSD pairwise comparison of secologanic acid production by CYP72A564 mutants.

Comparison	Difference in Means	SEM	q Value	Probability	Alpha	Significant?
WT<br>-P450	0.9838	0.09348	14.88	7.7E-07	0.05	Yes
H131F<br>-P450	-0.006220	0.09348	0.09412	1.00	0.05	No
H131F<br>WT	-0.9900	0.09348	14.98	7.1E-07	0.05	Yes
H132D<br>-P450	0.9516	0.09348	14.40	1.2E-06	0.05	Yes
H132D<br>WT	-0.03213	0.09348	0.4861	1.00	0.05	No
H132D<br>H131F	0.9579	0.09348	14.49	1.1E-06	0.05	Yes
H131F,H132D<br>-P450	0.02484	0.09348	0.3758	1.00	0.05	No
H131F,H132D<br>WT	-0.9589	0.09348	14.51	1.1E-06	0.05	Yes
H131F,H132D<br>H131F	0.03106	0.09348	0.4699	1.00	0.05	No
H131F,H132D<br>H132D	-0.9268	0.09348	14.02	1.7E-06	0.05	Yes
R270T<br>-P450	0.7681	0.09348	11.62	1.6E-05	0.05	Yes
R270T<br>WT	-0.2157	0.09348	3.263	0.31	0.05	No
R270T<br>H131F	0.7743	0.09348	11.71	1.5E-05	0.05	Yes
R270T<br>H132D	-0.1836	0.09348	2.777	0.48	0.05	No
R270T<br>H131F,H132D	0.7432	0.09348	11.24	2.4E-05	0.05	Yes
S324E<br>-P450	0.3929	0.09348	5.944	0.012	0.05	Yes
S324E<br>WT	-0.5908	0.09348	8.938	3.0E-04	0.05	Yes
S324E<br>H131F	0.3992	0.09348	6.038	0.011	0.05	Yes
S324E<br>H132D	-0.5587	0.09348	8.452	5.2E-04	0.05	Yes
S324E<br>H131F,H132D	0.3681	0.09348	5.569	0.019	0.05	Yes
S324E<br>R270T	-0.3751	0.09348	5.675	0.017	0.05	Yes

Table S8. Tukey's HSD pairwise comparison of secologanic acid production by CYP72A565 mutants.

Comparison	Difference in Means	SEM	q Value	Probability	Alpha	Significant?
WT<br>-P450	0.9886	0.04183	33.42	< 1E-9	0.05	Yes
H131F<br>-P450	0.004620	0.04183	0.1560	1	0.05	No
H131F<br>WT	-0.9839	0.04183	33.26	< 1E-9	0.05	Yes
H132D<br>-P450	0.5355	0.04183	18.10	6.4E-08	0.05	Yes
H132D<br>WT	-0.4530	0.04183	15.31	5.2E-07	0.05	Yes
H132D<br>H131F	0.5309	0.04183	17.95	6.9E-08	0.05	Yes
H131F,H132D<br>-P450	6.430E-04	0.04183	0.02174	1	0.05	No
H131F,H132D<br>WT	-0.9879	0.04183	33.40	< 1E-9	0.05	Yes
H131F,H132D<br>H131F	-0.003970	0.04183	0.1343	1	0.05	No
H131F,H132D<br>H132D	-0.5349	0.04183	18.08	6.4E-08	0.05	Yes
K270T<br>-P450	0.2373	0.04183	8.020	8.8E-04	0.05	Yes
K270T<br>WT	-0.7513	0.04183	25.40	1.5E-07	0.05	Yes
K270T<br>H131F	0.2326	0.04183	7.864	0.0011	0.05	Yes
K270T<br>H132D	-0.2983	0.04183	10.08	8.2E-05	0.05	Yes
K270T<br>H131F,H132D	0.2366	0.04183	7.998	9.0E-04	0.05	Yes
S324E<br>-P450	0.3897	0.04183	13.18	3.7E-06	0.05	Yes
S324E<br>WT	-0.5988	0.04183	20.24	1.2E-07	0.05	Yes
S324E<br>H131F	0.3851	0.04183	13.02	4.2E-06	0.05	Yes
S324E<br>H132D	-0.1458	0.04183	4.928	0.044	0.05	Yes
S324E<br>H131F,H132D	0.3891	0.04183	13.15	3.7E-06	0.05	Yes
S324E<br>K270T	0.1525	0.04183	5.155	0.033	0.05	Yes

Table S9. Tukey's HSD pairwise comparison of secologanin production by CYP72A564 mutants.

Comparison	Difference In Means	SEM	q Value	Probability	Alpha	Significant?
WT<br>-P450	0.9997	0.09585	14.74984	8.7E-7	0.05	Yes
H131F<br>-P450	1.8468	0.09585	27.24808	4.1E-8	0.05	Yes
H131F<br>WT	0.8471	0.09585	12.49824	6.9E-6	0.05	Yes
H132D<br>-P450	0.08288	0.09585	1.22285	0.97	0.05	No
H132D<br>WT	-0.91682	0.09585	13.52699	2.6E-6	0.05	Yes
H132D<br>H131F	-1.76392	0.09585	26.02523	3.9E-6	0.05	Yes
H131F,H132D<br>-P450	0.79978	0.09585	11.80012	1.4E-5	0.05	Yes
H131F,H132D<br>WT	-0.19992	0.09585	2.94972	0.41	0.05	No
H131F,H132D<br>H131F	-1.04702	0.09585	15.44796	4.7E-7	0.05	Yes
H131F,H132D<br>H132D	0.7169	0.09585	10.57727	4.8E-5	0.05	Yes
R270T<br>-P450	0.78529	0.09585	11.58633	1.7E-5	0.05	Yes
R270T<br>WT	-0.21441	0.09585	3.16352	0.34	0.05	No
R270T<br>H131F	-1.06151	0.09585	15.66176	3.8E-7	0.05	Yes
R270T<br>H132D	0.70241	0.09585	10.36347	6.0E-5	0.05	Yes
R270T<br>H131F,H132D	-0.01449	0.09585	0.2138	1.00	0.05	No
S324E<br>-P450	0.19991	0.09585	2.94955	0.41	0.05	No
S324E<br>WT	-0.79979	0.09585	11.80029	1.4E-5	0.05	Yes
S324E<br>H131F	-1.64689	0.09585	24.29853	1.7E-6	0.05	Yes
S324E<br>H132D	0.11703	0.09585	1.7267	0.87	0.05	No
S324E<br>H131F,H132D	-0.59987	0.09585	8.85057	3.3E-4	0.05	Yes
S324E<br>R270T	-0.58538	0.09585	8.63678	4.2E-4	0.05	Yes

Table S10. Tukey's HSD pairwise comparison of secologanin production by CYP72A565 mutants.

Comparison	Difference in Means	SEM	q Value	Probability	Alpha	Significant?
WT<br>-P450	0.99927	0.03613	39.10973	< 1E-9	0.05	Yes
H131F<br>-P450	1.20867	0.03613	47.30507	< 1E-9	0.05	Yes
H131F<br>WT	0.2094	0.03613	8.19534	7.1E-4	0.05	Yes
H132D<br>-P450	0.05168	0.03613	2.0226	0.78	0.05	No
H132D<br>WT	-0.94759	0.03613	37.08713	< 1E-9	0.05	Yes
H132D<br>H131F	-1.15699	0.03613	45.28247	< 1E-9	0.05	Yes
H131F,H132D<br>-P450	0.45526	0.03613	17.81791	7.4E-8	0.05	Yes
H131F,H132D<br>WT	-0.54402	0.03613	21.29182	1.3E-7	0.05	Yes
H131F,H132D<br>H131F	-0.75341	0.03613	29.48717	3.9E-8	0.05	Yes
H131F,H132D<br>H132D	0.40358	0.03613	15.79531	3.4E-7	0.05	Yes
K270T<br>-P450	0.35981	0.03613	14.08213	1.5E-6	0.05	Yes
K270T<br>WT	-0.63947	0.03613	25.02759	1.4E-7	0.05	Yes
K270T<br>H131F	-0.84886	0.03613	33.22294	< 1E-9	0.05	Yes
K270T<br>H132D	0.30813	0.03613	12.05954	1.1E-5	0.05	Yes
K270T<br>H131F,H132D	-0.09545	0.03613	3.73577	0.19	0.05	No
S324E<br>-P450	0.19876	0.03613	7.77905	1.2E-3	0.05	Yes
S324E<br>WT	-0.80051	0.03613	31.33067	< 1E-9	0.05	Yes
S324E<br>H131F	-1.00991	0.03613	39.52602	< 1E-9	0.05	Yes
S324E<br>H132D	0.14708	0.03613	5.75646	0.015	0.05	Yes
S324E<br>H131F,H132D	-0.2565	0.03613	10.03885	8.6E-5	0.05	Yes
S324E<br>K270T	-0.16105	0.03613	6.30308	7.6E-3	0.05	Yes

Table S11. Tukey's HSD pairwise comparison of secologanic acid production by CYP72A564, CYP72A565, and the SLS, SLAS common ancestor.

Comparison	Difference in Means	SEM	q Value	Probability	Alpha	Significant?
72A564 -P450	0.99635	0.15116	9.32137	7.75946E-4	0.05	Yes
72A565 -P450	0.86962	0.15116	8.1358	0.00191	0.05	Yes
72A565 72A564	-0.12672	0.15116	1.18557	0.83513	0.05	No
Ancestor -P450	0.02201	0.15116	0.20591	0.9988	0.05	No
Ancestor 72A564	-0.97434	0.15116	9.11546	9.02481E-4	0.05	Yes
Ancestor 72A565	-0.84761	0.15116	7.92989	0.00226	0.05	Yes

Table S12. Tukey's HSD pairwise comparison of secologanin production by CYP72A564, CYP72A565, and the SLS, SLAS common ancestor.

Comparison	Difference in Means	SEM	q Value	Probability	Alpha	Significant?
72A564 -P450	0.99427	0.18904	7.43804	0.00338	0.05	Yes
72A565 -P450	0.52359	0.18904	3.91694	0.09208	0.05	No
72A565 72A564	-0.47068	0.18904	3.52111	0.13634	0.05	No
Ancestor -P450	0.04279	0.18904	0.32011	0.99558	0.05	No
Ancestor 72A564	-0.95148	0.18904	7.11794	0.00444	0.05	Yes
Ancestor 72A565	-0.4808	0.18904	3.59683	0.12654	0.05	No

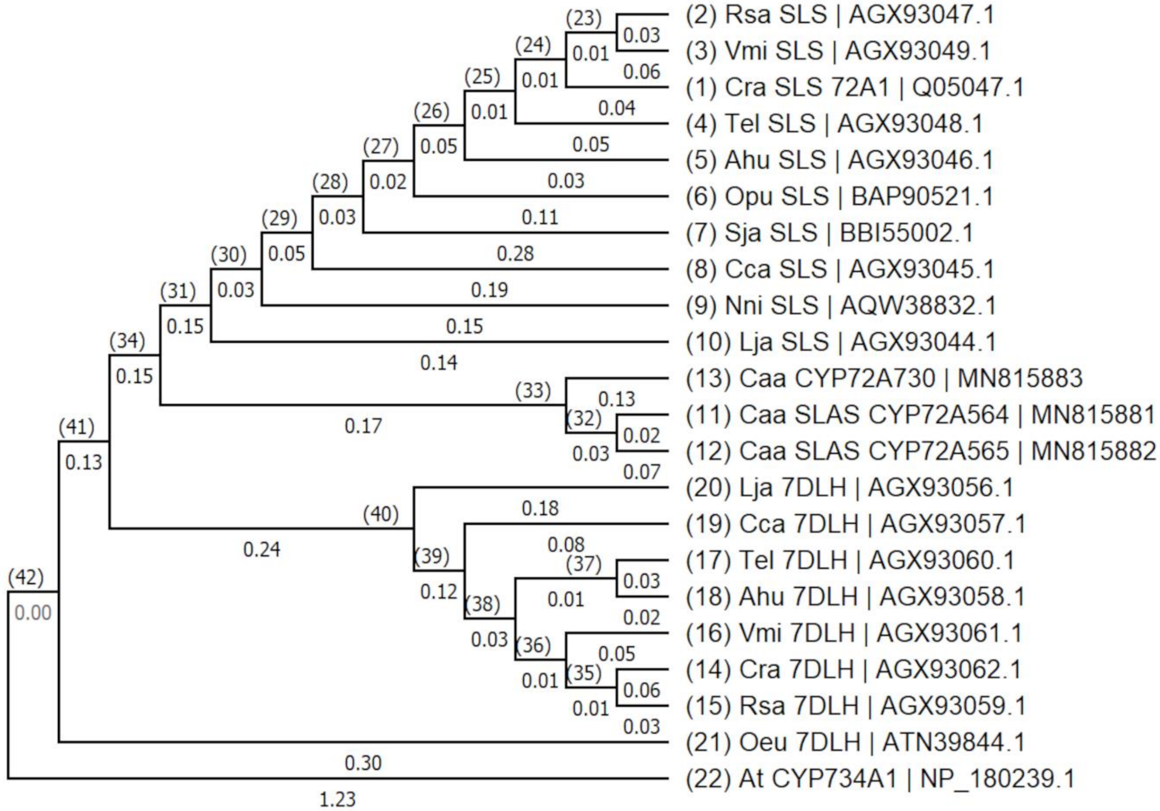

Figure S1. Bootstrap consensus tree containing node identifications (in parentheses) and branch lengths for the ancestral sequence reconstruction.

>SLS,SLAS_Ancestor Node_34
MEMDLLYKSIAASVLVALLVWAWRVLNWAWFTPKRLEKRLRQQGFKGNPYRLLVGDLKESSMMLKEAMSKPIPFSN
DIVPRIMPHIVKTIETYGKNSFTWMGRMPRIHIMEPELIREVLTNSNKFQKNHHAHNPLTKFLLTGIGSLEGDKWA
KHRRIISPAFHLEKLKTMLPAFYISYDDLLSKWEKVASTEGSVEVDVFPTFDTLTSDVISRVAFGSNYEEGGKIFQ
LLKELMDLTIEVMRSVYIPGWSFLPTKRNQRMREIDKEIRERLSNIINKRVKAMKAGEPSGDDLLGVLLESNFKEI
QRQGNKKNAGMSIEDVIEECKLFYFAGQETTGILLTWTMVLLSRHPEWQERAREEVLQVFGNGKPDFDRLNHLKIV
SMILYEVLRLYPPVIELTKVVHEETKLGNLTIPAGVQLMMPTILLHRDKEIWGDDAMEFNPGRFAEGVAKATKSQV
SYIPFSWGPRICIGQNFALLQAKMALAMILQRFSFDLSPSYAHAPFTVLTLQPQYGAHVIFRKLKS

Key
100%
95-100%
90-95%
80-90%
70-80%
60-70%
0 -60%

Figure S2. Amino acid sequence of SLS, SLAS common ancestor color coded by probability from ancestral sequence reconstruction.

```
Cra_CYP72A1         MEMDMDTIRKAIAATIFALVMAWAWRVLDWAWFTPKRIEKRLRQQGFRGNPYRFLVGDVKESGKMHQEAL    70
Ancestor            --MEMDLLYKSIAASVLVALLVWAWRVLNWAWFTPKRLEKRLRQQGFKGNPYRLLVGDLKESSMMLKEAM    68
Caa_CYP72A564       MEIQMDVLYKSIAASVAVVFLVYAWKMLNWAYLTPKRIEKCLRKQGFKGNSYRLLVGDLKESSMMLKEAM    70
Caa_CYP72A565       MEIQMDVLYKSIAASVAVVFLVYAWKMLNWAYLTPKRIEKCLRKQGFKGNSYRLLVGDLKESSMMLKETM    70
                      ::** : *:***:: . .:.:**::*:**::****:** **:***:** **:****:***. * :*::

Cra_CYP72A1         SKPMEFNNDIVPRLMPHINHTINTYGRNSFTWMGRIPRIHVMEPELIKEVLTHSSKYQKNFDVHNPLVKF   140
Ancestor            SKPIPFSNDIVPRIMPHIVKTIETYGKNSFTWMGRMPRIHIMEPELIREVLTNSNKFQKNHHAHNPLTKF   138
Caa_CYP72A564       SKPIPVSQDIVQRLMPHVVKTIQTYGKNSFTWIGRMPRVHIMEPELIKDILANHNNFQKNHHAYSPLTKF   140
Caa_CYP72A565       SKPINVSEDIVQRVMPHVIKTIDTYGKNSFTWIGRMPRVHIMEPDLIKDILANHNDFMKNHHAYNPLTKF   140
                    ***: ..:*** *:***: :**:***:*****:**:**:*:***:**:::*:: ..: **...:.**.**

Cra_CYP72A1         LLTGVGSFEGAKWSKHRRIISPAFTLEKLKSMLPAFAICYHDMLTKWEKIAEKQGSHEVDIFPTFDVLTS   210
Ancestor            LLTGIGSLEGDKWAKHRRIISPAFHLEKLKTMLPAFYISYDDLLSKWEKVASTEGSVEVDVFPTFDTLTS   208
Caa_CYP72A564       LLTGIGSLEGEKWAKHRRIISPSFHLEKLKTMLPAFYVSYDELLGKWERESSTKGSVEVDLFPTFDTLTS   210
Caa_CYP72A565       LLTGIGSLEGDKWAKHRRIISPSFHLEKLKTMLPAFYVSYDDLLTKWEQQCSSKGSVEIDLFPTFDTLTS   210
                    ****:**:** **:********:* *****:***** :.*.::* ***: ...:** *:*:*****.***

Cra_CYP72A1         DVISKVAFGSTYEEGGKIFRLLKELMDLTIDCMRDVYIPGWSYLPTKRNKRMKEINKEITDMLRFIINKR   280
Ancestor            DVISRVAFGSNYEEGGKIFQLLKELMDLTIEVMRSVYIPGWSFLPTKRNQRMREIDKEIRERLSNIINKR   278
Caa_CYP72A564       DVISRVAFGSSYGEGGRIFILLKELMDLTVDVMRSVYVPGWSLLPTKRNQRMREVDREIRERLSGIINSR   280
Caa_CYP72A565       DVISRVAFGSSYGEGGRIFILLKELMDLTVDVMRSVYVPGSSFLPTKRNNRMREVDGEIKDRLSGIINSR   280
                    ****:*****.* ***:** *********:: **.**:** * ******:**:*:: ** : *  ***.*

Cra_CYP72A1         MKALKAGEPGEDDLLGVLLESNIQEIQKQGNKKDGGMSINDVIEECKLFYFAGQETTGVLLTWTTILLSK   350
Ancestor            VKAMKAGEPSGDDLLGVLLESNFKEIQRQGNKKNAGMSIEDVIEECKLFYFAGQETTGILLTWTMVLLSR   348
Caa_CYP72A564       VKAMKAGEPSGDDLLGTLLESNFREIERLGNKKNAGMSIEDVISECKLFYFAGQETTGILLTWTCVLLSR   350
Caa_CYP72A565       VKAMKAGEPSGEDLLGTLLESNFKEIERLGNKKNAGMSIEDVISECKLFYFAGQETTGILLTWTCVLLSR   350
                    :**:*****. :****.*****::**:: ****:.****:***.**************:***** :***:

Cra_CYP72A1         HPEWQERAREEVLQAFGKNKPEFERLNHLKYVSMILYEVLRLYPPVIDLTKIVHKDTKLGSYTIPAGTQV   420
Ancestor            HPEWQERAREEVLQVFGNGKPDFDRLNHLKIVSMILYEVLRLYPPVIELTKVVHEETKLGNLTIPAGVQL   418
Caa_CYP72A564       HPEWQERAREEIFQVFGNGKLDFDRVQGLKIVPMILYEVLRLYPPVIELTKVTYEEQKLGNLTIPAGVQL   420
Caa_CYP72A565       HPEWQERAREEIFQVFGNGKVDFDRVQNLKIVPMILYEVLRLYPPVIELTKVTYEEQKLGNLTIPAGVQL   420
                    ***********::*.**:.* :*:*:: ** * **************:***:.::: ***. *****.*:

Cra_CYP72A1         MLPTVMLHREKSIWGEDAMEFNPMRFVDGVANATKNNVTYLPFSWGPRVCLGQNFALLQAKLGLAMILQR   490
Ancestor            MMPTILLHRDKEIWGDDAMEFNPGRFAEGVAKATKSQVSYIPFSWGPRICIGQNFALLQAKMALAMILQR   488
Caa_CYP72A564       MMPSILLHRDKEMWGDDATEFNPGRFAEGVAKAVKSPFFYIPFSWGPRICVGQNFALLQAKMALAMILQR   490
Caa_CYP72A565       MMPSILLHRDQEMWGADSKEFNPGRFADGISKAVKSPFFYIPFSWGPRICVGQNFALLQAKMALTMILQR   490
                    *:*:::***::.:** *: **** **.:*:::*.*. . *:*******:*:**********:.*:*****

Cra_CYP72A1         FKFDVAPSYVHAPFTILTVQPQFGSHVIYKKLES   524
Ancestor            FSFDLSPSYAHAPFTVLTLQPQYGAHVIFRKLKS   522
Caa_CYP72A564       FSFDLSPTYAHAPFTVLTLQPQHGAQVIFRRLKC   524
Caa_CYP72A565       FTFDLSPTYAHAPFTVLTLQPQHGAQVVFRKIKC   524
                    *.**::*:*.*****:**:***.*::*::::::.
```

Figure S3. Multiple sequence alignment of *Catharanthus* CYP72A1, *Camptotheca* CYP72A564 and CYP72A565, and their common ancestor.

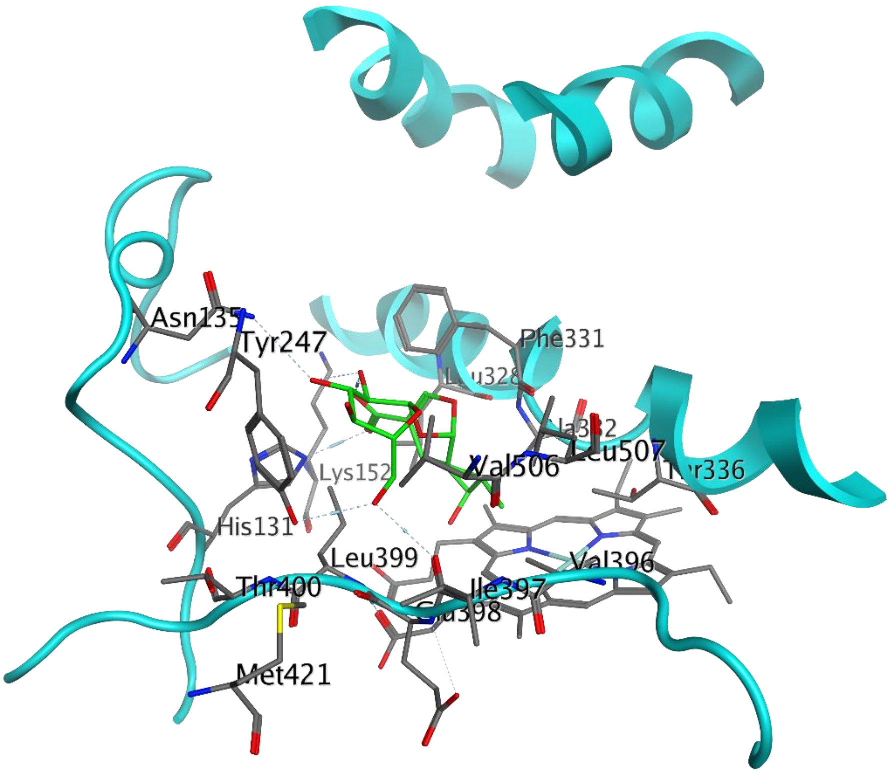

Figure S4. Amino acid contacts of docked loganic acid in homology model of SLS, SLAS common ancestor.
Residues within 4.5 Å of loganic acid (green) are shown with SRSs 1-5 shown in ribbon (cyan).

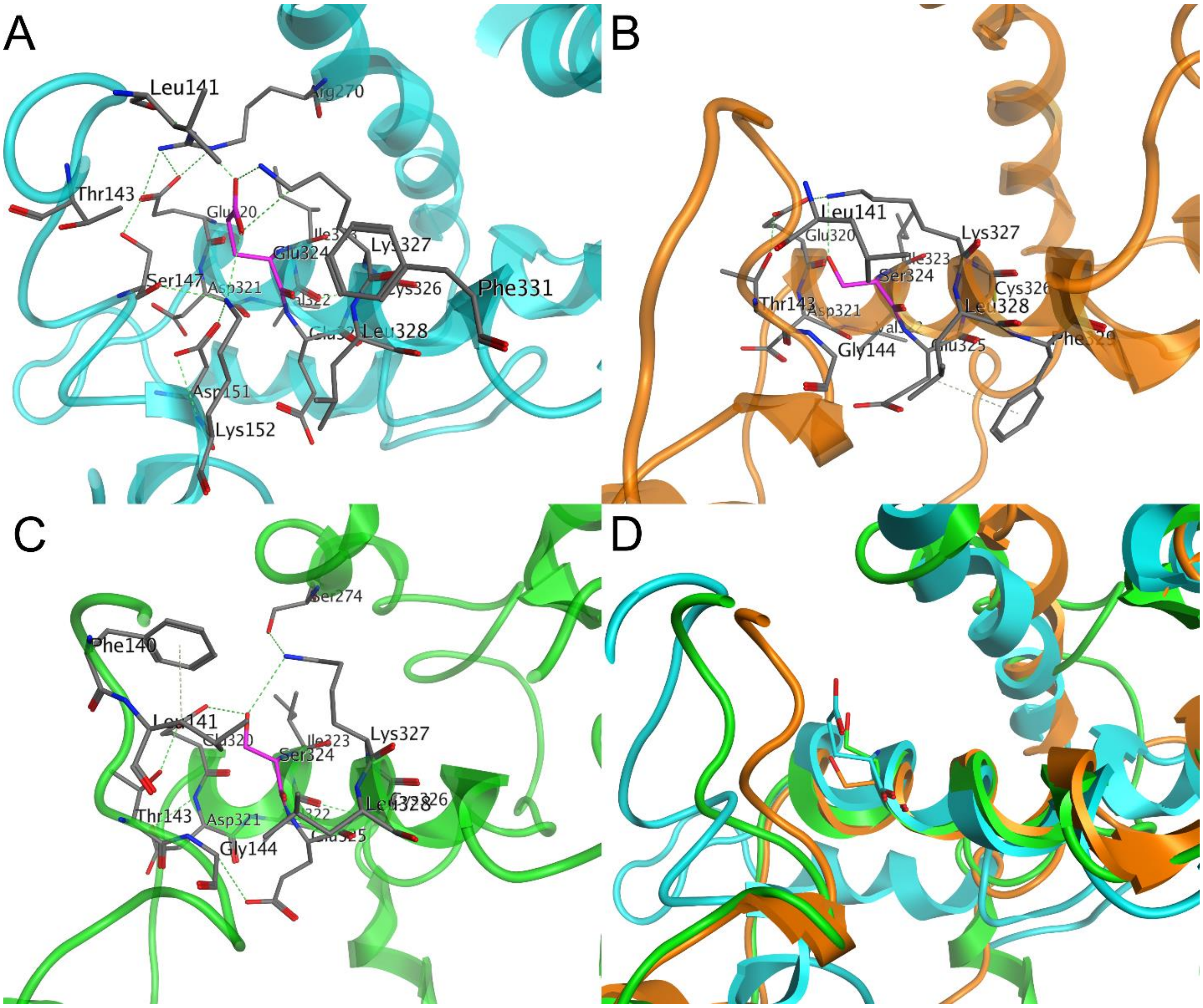

Figure S5. Hydrogen bonding networks around site 324 in homology models.
All residues within 4.5 Å of the amino acid at site 324 (magenta) are shown for the SLS, SLAS ancestor (A), CYP72A564 (B), CYP72A565 (C). (D) A ribbon overlay of all three models with site 324 shown. Hydrogen bonds are shown as green dotted lines.

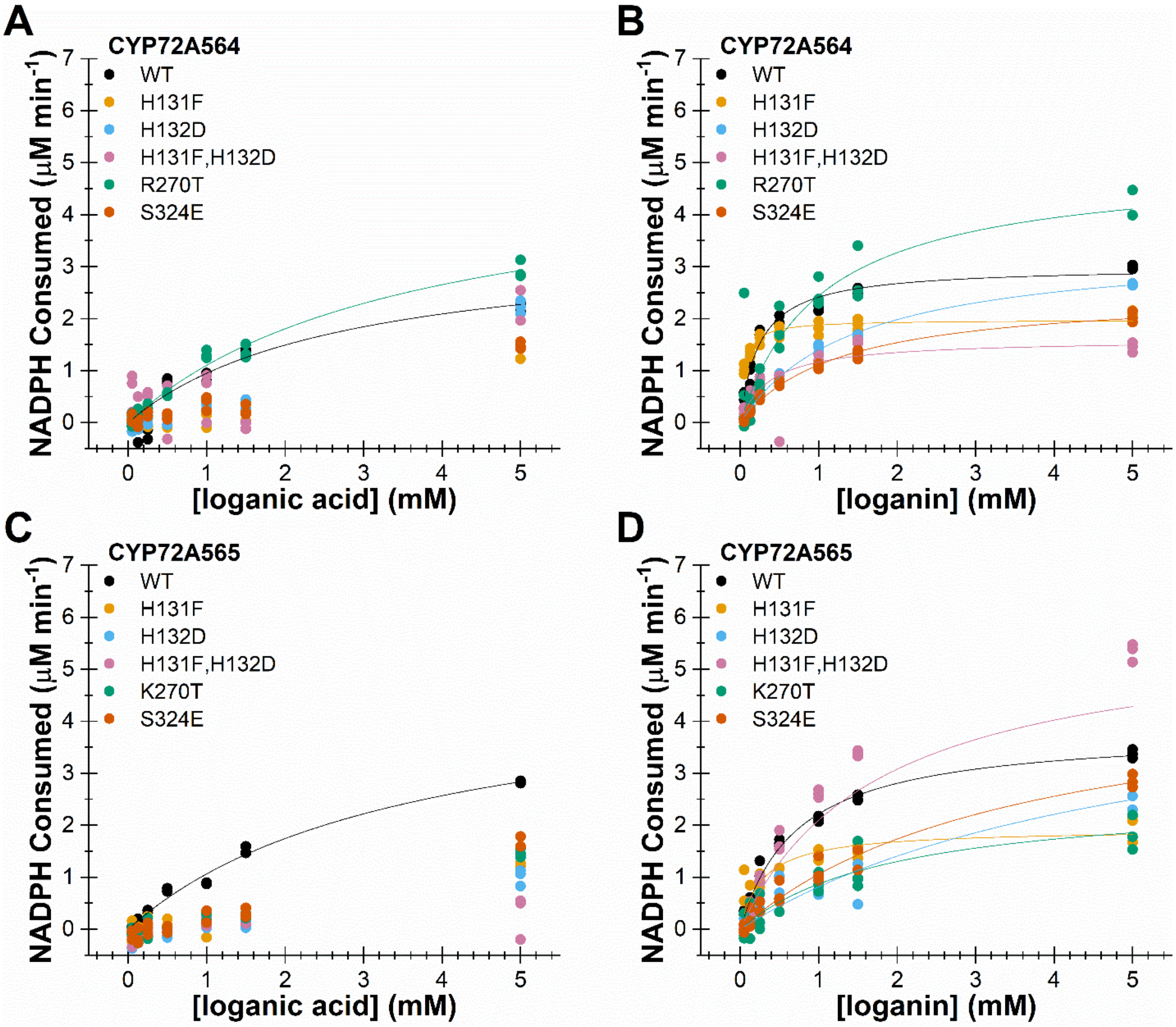

Figure S6. Steady-state kinetics of loganic acid and loganin turnover for all CYP72A564 and CYP72A565 mutants.

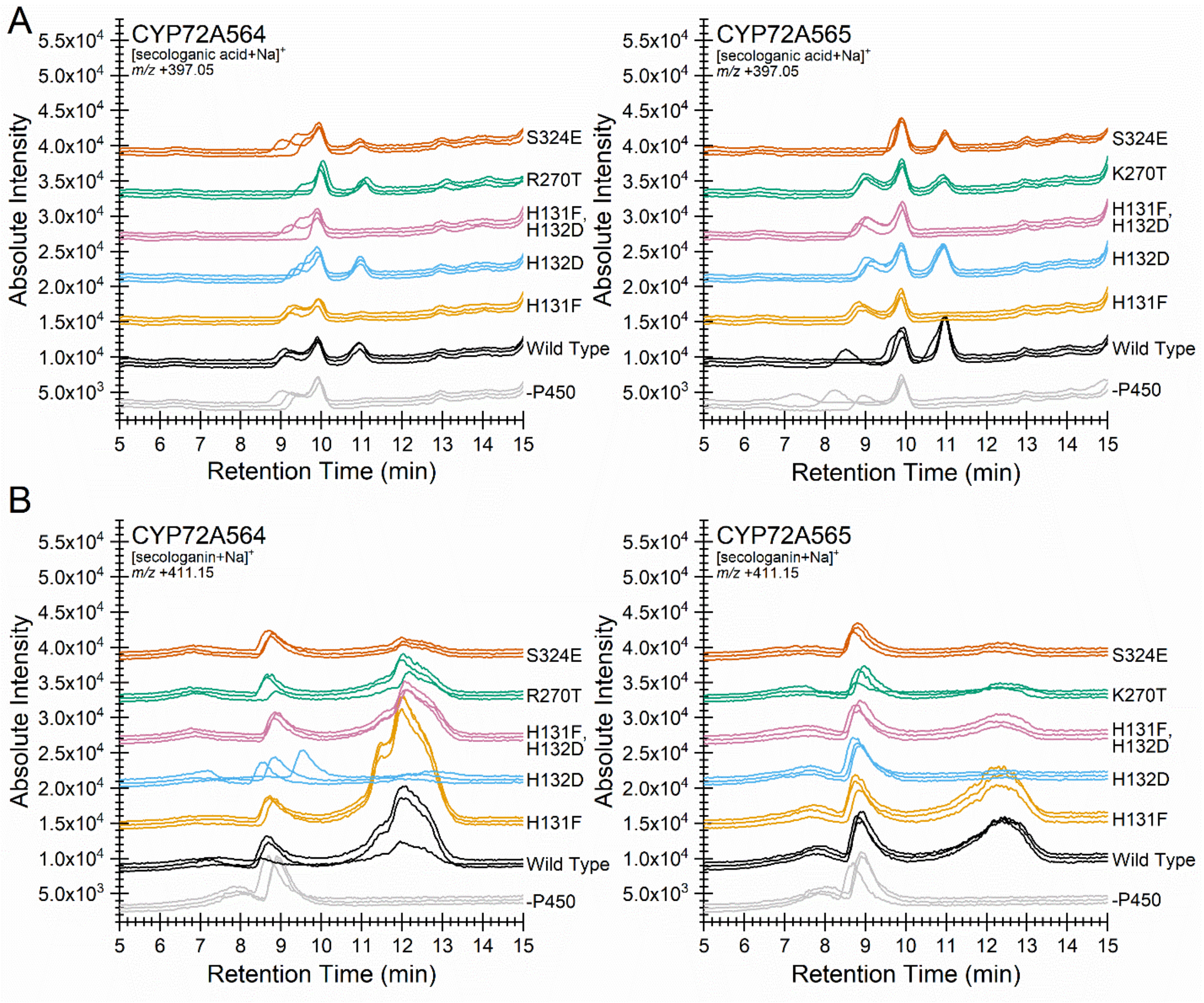

Figure S7. Extracted ion chromatograms of secoiridoids from *in vitro* reactions.
Selective ion monitoring at *m/z* +397.05 [secologanic acid+Na]$^+$ (A) and *m/z* +411.15 [secologanin+Na]$^+$ (B) were used to assay secoiridoid production for CYP72A564 (left) and CYP72A565 (right) mutants. Reactions included 250. nM P450, 0.5 mU µL^{-1} *Camptotheca* CPR1, 1 mM DLPC, 250. µM substrate, 500 µM NADPH, 1.00 mM glucose-6-phosphate, and 0.1 U µL^{-1} G6PDH in 100. mM $NaPO_4$ (pH 7.5). Retention times: loganic acid, 10.0 min; secologanic acid, 11.0 min; loganin, 8.8 min; secologanin, 12.5 min.

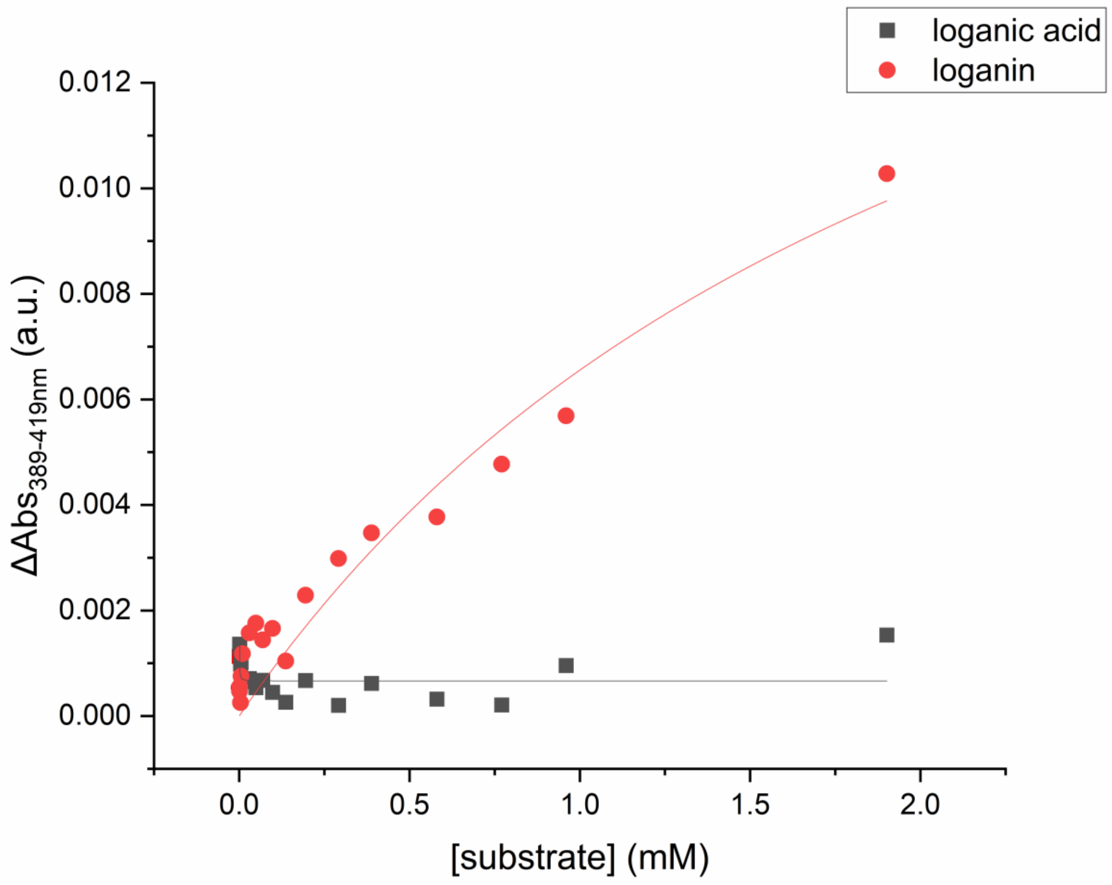

Figure S8. Binding isotherm from SLS, SLAS common ancestor Type I binding spectra.
Binding isotherms derived by plotting the difference between the peak (389 nm) and trough (419 nm) of the difference spectra against the substrate concentration using OriginPro 2019. Table S11 records the fit parameters.
